# Structural and functional insights into the lipid regulation of human anion exchanger 2

**DOI:** 10.1038/s41467-024-44966-0

**Published:** 2024-01-26

**Authors:** Weiqi Zhang, Dian Ding, Yishuo Lu, Hongyi Chen, Peijun Jiang, Peng Zuo, Guangxi Wang, Juan Luo, Yue Yin, Jianyuan Luo, Yuxin Yin

**Affiliations:** 1 Institute of Systems Biomedicine, Department of Pathology, Beijing Key Laboratory of Tumor Systems Biology, Peking-Tsinghua Center for Life Sciences, School of Basic Medical Sciences, Peking University Health Science Center, Beijing, 100191 China; 2 Institute of Precision Medicine, Peking University Shenzhen Hospital, Shenzhen, 518036 China; 3 Department of Pharmacology, School of Basic Medical Sciences, Peking University Health Science Center, Beijing, China; 4 Department of Medical Genetics, School of Basic Medical Sciences, Peking University Health Science Center, Beijing, China

**Keywords:** Cryoelectron microscopy, Carrier proteins, Diseases, Permeation and transport

## Abstract

Anion exchanger 2 (AE2) is an electroneutral Na^+^-independent Cl^-^/HCO_3_^-^ exchanger belongs to the SLC4 transporter family. The widely expressed AE2 participates in a variety of physiological processes, including transepithelial acid-base secretion and osteoclastogenesis. Both the transmembrane domains (TMDs) and the N-terminal cytoplasmic domain (NTD) are involved in regulation of AE2 activity. However, the regulatory mechanism remains unclear. Here, we report a 3.2 Å cryo-EM structure of the AE2 TMDs in complex with PIP_2_ and a 3.3 Å full-length mutant AE2 structure in the resting state without PIP_2_. We demonstrate that PIP_2_ at the TMD dimer interface is involved in the substrate exchange process. Mutation in the PIP_2_ binding site leads to the displacement of TM7 and further stabilizes the interaction between the TMD and the NTD. Reduced substrate transport activity and conformation similar to AE2 in acidic pH indicating the central contribution of PIP_2_ to the function of AE2.

## Abstract (web summary)

Anion exchanger 2 (AE2), a widely expressed Cl- /HCO3 - exchanger, participates in the regulation of intracellular pH. Here, the authors present the structures of AE2 and uncover the regulatory mechanism of PIP2.

## Introduction

The intracellular pH of eukaryotic cells is stringently regulated to maintain the structure and function of proteins that have critical roles in membrane excitability, cytoskeletal dynamics, and vesicle trafficking^1^. Cells employ a complex intrinsic buffering capacity and a variety of ion carriers at the membrane, mainly Na^+^/H^+^ exchangers (SLC9 family) and bicarbonate-transporters family (SLC4 family), to limit the intracellular pH within a narrow physiological range^1^. The *SLC4* family consists of ten genes that encode integral membrane proteins, which can be divided into three groups: Cl^−^/ HCO_3_^−^ exchangers (AE subfamily), Na^+^-coupled HCO_3_^−^ transporters (NBCe1, NBCe2, NBCn1, NBCn2, NDCBE), and a H^+^(OH^−^) transporter BTR1^2^. AE1-3 (*SLC4A1-3*) are Na^+^-independent monovalent anion exchangers, crucial in conveying CO_2_ from systemic tissues to the lungs, gastric acid secretions^3^, and biliary base secretions^4^. AE2 is approximately 55%-56% identical to AE1 and AE3 at the amino acid level and it is the most widely expressed AE found at the basolateral membrane of most epithelia^2,5^. Alternative splicing of the *SLC4A2* gene gives rise to three AE2 isoforms that differ in their N-terminal sequences^6^. The promoter of *SLC4A2a* is active in most tissues indicative of its housekeeping function^7^. In contrast, expression of AE2b is restricted to epithelia of stomach, liver, kidney and lung tissues, and AE2c is only expressed in the stomach, which suggests tissue-specific functions^3,7^. AE2 serves as the major Cl^−^/HCO_3_^−^ exchanger at the basolateral membrane in gastric parietal cells where it participates in gastric acid secretion^8^. An achlorhydric phenotype of *SLC4A2*^−/−^ mice further confirms the impairment of gastric acid secretion caused by the loss of AE2^3^. *SLC4A2* knockdown experiments in mammalian cholangiocytes have implicated AE2 as the main effector in biliary HCO_3_^−^ secretion^4^ and furthermore, *SLC4A2* knockdown mice exhibited typical symptoms of primary biliary cholangitis (PBC), including generation of antimitochondrial antibodies (AMA), portal inflammation and damaged interlobular bile ducts^9–11^. AE2 point mutations leading to AE2 dysfunction have also been reported in a patient with autosomal recessive osteopetrosis^12^.

AE2 is regulated by environmental pH, NH_4_^+^, hypertonicity and calmidazolium^13–17^. The transmembrane domains (TMD) and the cytoplasmic N-terminal domain (NTD) of AE2 are both involved in sensing intracellular pH, NH_4_^+^ and hypertonicity, while sensing of extracellular pH (pHe) and the inhibitory effect of the small molecule calmidazolium relies mainly on the TMD of AE2^18–20^. Several structures of SLC4 family proteins have been solved in recent years, including AE1^21–24^, AE2^25^, NBCe1^26^, NDCBE^27^, and BTR1^28^. Recently determined structures of AE2 in different states showed an interlocking mechanism of interactions between the TMDs and the NTDs and identified a self-inhibitory C-terminal loop^25^. The structure of a human ankyrin-1 complex revealed an interaction between AE1 and the signaling lipid, phosphatidyl 4,5-biphosphate (PIP_2_) molecules^23^, which had been previously reported as activators of NBCe1^29–31^ and NBCn1^32^. Structures of BTR1 showed that disruption of PIP_2_ binding site leads to inward-facing conformation similar to BTR1 in acidic pH^28^. However, the detailed pH sensing mechanism of AE2 and whether PIP_2_ participates in the regulation of AE family member activities have yet to be studied.

In this work, we determine the cryo-EM structures of human AE2 TMDs in complex with its activating cofactor PIP_2_ as well as full-length mutant AE2 in the absence of PIP_2_ in an inward-facing state. These structures together with functional analysis reveal the mechanism of how PIP_2_ modulates TMD and NTD coupling and the transport of substrates by AE2.

## Results

### Overall structure of AE2 in complex with PIP_2_

**Fig. 1:**
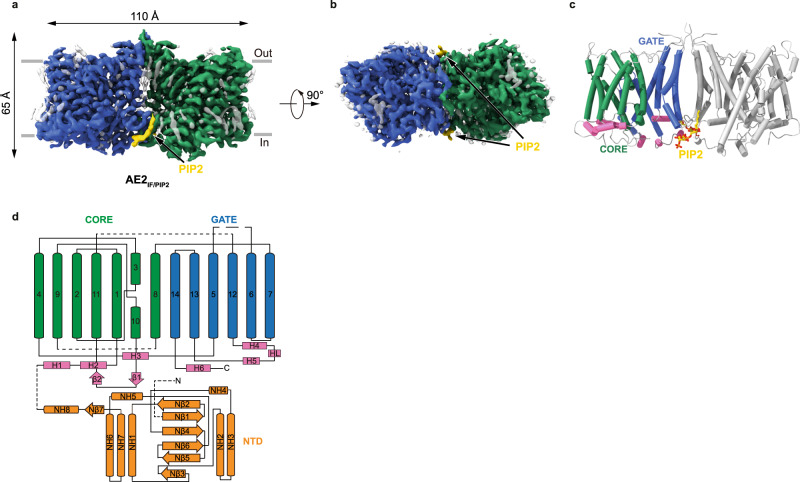
Structure of AE2 in complex with PIP_2_. **a**, **b** Cryo-EM density map of AE2_IF/PIP2_, viewed from the side (**a**) and the cytoplasmic side (**b**). The approximate position of the lipid bilayer is indicated by gray bars. The two protomers, PIP_2_ and lipid-like densities are colored in green, blue, yellow and gray, respectively. **c** Structural model of AE2 shown in cylinder representation. The core domain, gate domain, and intracellular helices of one transmembrane domain (TMD) are colored in green, blue and pink, respectively. The other protomer is colored in gray. **d** Cartoon illustrating the topology of AE2. The N-terminal cytoplasmic domain (NTD, amino acids 320-431 and 503–586) is colored in orange and other structural features are colored as in panel **c**.

Full-length canonical human AE2 (isoform 1), fused with a C-terminal green fluorescence protein (GFP) and an affinity tag, was expressed in HEK293F cells^33^. Recombinant AE2 protein was extracted from membranes using lauryl maltose neopentyl glycol (LMNG) and cholesteryl hemisuccinate (CHS) and then LMNG exchanged with glycol-diosgenin (GDN) by size exclusion chromatography. Purified proteins were then subjected to cryo-EM analysis and a structure of the AE2 TMDs in an inward-facing conformation at 3.2 Å resolution was determined (Supplementary Fig. 1). The TMDs of AE2 form a homodimer with two protomers in the same conformation and occupy a 3D space of approximately 110 Å × 65 Å × 65 Å (Fig. 1a, b). Each protomer is comprised of 14 TM helices, resembling the arrangement of UraA^34^, with a core domain formed by TMs 1-4 plus 8-11, and a gate domain formed by TMs5-7 plus 12-14 (Fig. 1c, d), similar to other SLC4 family proteins^21–23,26,35^. The density corresponding to the cytoplasmic NTD could not be identified because of its flexibility suggesting a weak interaction between the NTD and TMD in this conformation (NTD located away from TMD) (Supplementary Fig. 2a). Similar to the NTD, partial densities of extracellular loop (EL) 3, EL4, EL6 and the cytoplasmic C-terminus were less well-solved (again due to the flexibility of these regions) and were not included in our models. The dimer interface between the TMDs was formed by hydrophobic interactions between TM6, IL4, and IL7 (Supplementary Figs. 2b, c), and by hydrogen bonds and electrostatic interactions between EL3 and EL4 (Supplementary Fig. 2d). The disulfide bond formed between C855 of the two protomers stabilized the dimer interface (Supplementary Fig. 2d) and as such, may affect the sensitivity of AE2 to pHe as well as AE2 substrate transport activity^36^. The substrate coordination site was accessible from the cytoplasmic side of AE2 with a density likely corresponding to Cl^−^ (Supplementary Fig. 2e). The high-resolution map allowed us to unambiguously identify density features corresponding to PIP_2_ molecules with incompletely visualized and defined acyl chains (Supplementary Fig. 2f), located at the TMD dimer interface (Fig. 1a–c). Therefore, we designated this structure as the AE2_Inward Facing/with PIP2_ (AE2_IF/PIP2_) state.

### PIP_2_ binding site at the dimer interface of AE2

**Fig. 2:**
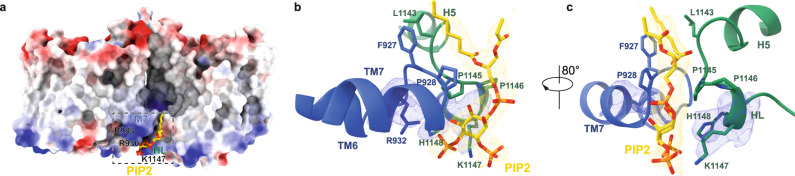
PIP_2_ binding site in AE2. **a** Surface representation of AE2 colored according to its electrostatic surface potentials. PIP_2_ is shown as sticks and colored in yellow. TM7 and HL are indicated in Fig. 1d. **b**, **c** Close-up view of the PIP_2_ binding site. Densities of PIP_2_ and amino acids R932, K1147 and H1148 are shown in mesh. PIP_2_ and the two protomers of AE2 are colored in yellow, blue and green, respectively. PIP_2_ and the amino acids that interact with PIP_2_ are shown as sticks.

Densities of PIP_2_ were observed between intracellular loop 4 (IL4) of one protomer and IL7 of the other protomer (Fig. 2), consistent with the reported binding sites of PIP_2_ in AE1^23^. Two lipid-like densities were found near PIP_2_ at positions corresponding to cholesterol in AE1 (8CT3) and CHS in AE2 (8GVH); however, poor resolution hindered the determination of their identities (Supplementary Fig. 2g). Densities of PIP_2_ were recognized in the dimer interface in previous AE1 structures^22^ and the density near PIP_2_, which corresponded to cholesterol in AE1/8CT3, did not show clear features of cholesterol (Supplementary Fig. 2h)^22^. Positively charged residues contributed by TM7 and lipid binding helix (HL) between TM12 and TM13 formed the PIP_2_ binding sites (Figs. 1d and 2a). Specifically, positive residues R930, R932 located on TM7 of one protomer and K1147, H1148 in HL of the other protomer constitute the binding pocket and interact with the phosphate groups on the inositol ring of PIP_2_ (Fig. 2b, c). The acyl chains of PIP_2_ hydrophobically interact with non-polar residues F926, F927, P928 on IL4 and L1143, and P1145 and P1146 in the loop connecting HL to helix 5 (H5) (Figs. 1d and 2b, c). Although PIP_2_ molecules were previously identified in the erythrocyte ankyrin-1 complex^23^, the molecular action of PIP_2_ on AE1 substrate transport activity remains unclear.

**Fig. 3:**
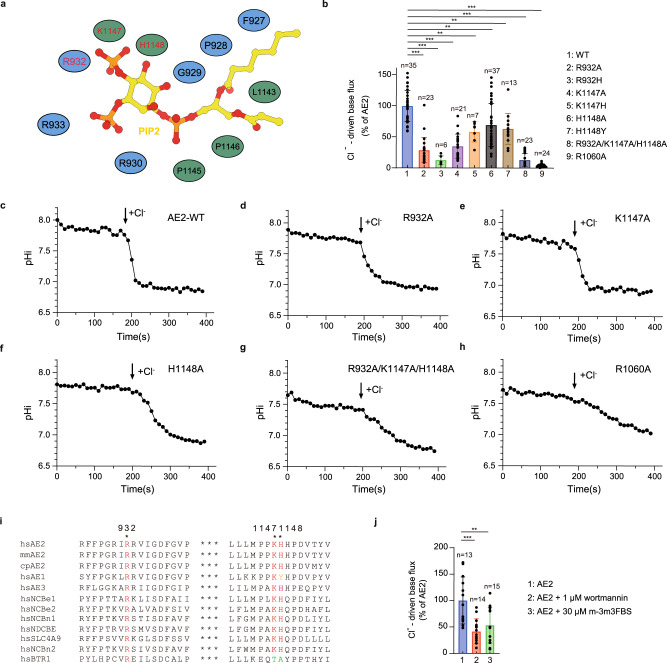
Interactions between PIP_2_ and AE2. **a** A cartoon model of the interactions between PIP_2_ (yellow) and the two protomers (blue and green) of AE2. PIP_2_ is shown as sticks and interacting residues on AE2 are shown as ovals. Mutated residues in (**b**) are labeled in red. **b** Cl^−^-driven base transport analysis of mutant AE2 activity normalized to wild-type AE2 activity at 2.78 × 10^−7 ^M/s. Data of AE2-WT (*n* = 35 biologically independent experiments), R932A (*n* =*n* = 23, *p* = 2.66 × 10^−15^), R932H (*n* = 6, *p* = 5.55 × 10^−16^), K1147A (*n* = 21, *p* = 3.51 × 10^−13^), K1147H (*n* = 7, *p* = 0.0032), H1148A (*n* = 37, *p* = 0.0021), K1148Y (*n* = 13, *p* = 0.0060), R932A/K1147A/H1148A (*n* = 23, *p* < 0.01 × 10^−16^) and R1160A (*n* = 24, *p* = 4.00 × 10^−15^) are shown as means ± SD of n ≥ 3 biologically independent experiments and significance determined by one-way ANOVA and post hoc Dunnett’s test (*p < 0.05, **p < 0.01, ***p < 0.001). **c**–**h** Typical traces of wild-type (**c**) and mutant (**d**–**h**) AE2 function. **i** Sequence alignments of the PIP_2_ binding site of *Homo sapiens* AE2 (hsAE2), *Mus musculus* AE2 (mmAE2), *Cavia porcellus* AE2 (cpAE2), and AE1 (SLC4A1), AE3 (SLC4A3), NCBe1 (SLC4A4), NBCe2 (SLC4A5), NBCn1 (SLC4A7), NDCBE (SLC4A8), SLC4A9, NBCn2 (SLC4A10) and BTR1 (SLC4A11) from *Homo sapiens* by PROMALS3D. The amino acids in AE2 that are important for interaction with PIP_2_ are colored in red and marked with asterisks above. **j** Effect of 1 μM wortmannin (Phosphoinositide kinase inhibitor) and 10 μM m-3m3FBS (phospholipase activator) on AE2 activity as measured by Cl^−^-driven base transport analysis. Data of AE2 (*n* = 13 biologically independent experiments), AE2 with wortmannin (*n* = 14, *p* = 0.0002) and AE2 with m-3m3FBS (*n* = 15, *p* = 0.002) are shown as means ± SD of *n* ≥ 3 biologically independent experiments and statistical significance determined by one-way ANOVA and post hoc Dunnett’s test (*p < 0.05, **p < 0.01, ***p < 0.001).

To investigate the functional impact of PIP_2_ on AE2, we performed mutagenesis analysis of residues designed to disrupt the PIP_2_ binding sites (Fig. 3a). As PIP_2_ functions as a plasma membrane recognition signal for vesicle docking and recruitment of many proteins^37^, we measured the effects of these mutations on AE2 plasma membrane localization prior to determining their effects on AE2 substrate transport activity (Supplementary Fig. 3). The mutation of hAE2 R1138C, which corresponds to mAE2 mutation R1134C lacking cell surface expression^38^, almost completely abolished AE2 plasma membrane localization (Supplementary Fig. 3j, k). Charge-neutralizing mutations of R932 and H1148 slightly reduced the plasma membrane localization of hAE2 (Supplementary Fig. 3c, d, g, k). Cells transfected with hAE2 with mutations in the PIP_2_ binding site or R1060A tended to be stabilized at a more acidic intracellular pH relative to wild-type hAE2 after balancing with Cl^−^ free buffer. In addition to reducing membrane localization, the R932 and H1148 mutations significantly decreased the Cl^−^-driven base efflux activity of hAE2 (Figs. 3b, d, f). Mutations of K1147 to less positively charged residues also decreased hAE2 activity (Fig. 3b, e). The R932A/K1147A/H1148A triple mutation had a similar effect on AE2 activity as the R1060A mutation, which disrupted the substrate coordination site (Fig. 3g, h)^38^. PIP_2_ depletion in intact cells also reduced the activity of wild-type hAE2 (Fig. 3j). Decreased hAE2 activity and plasma membrane localization induced by mutations in the PIP_2_ binding site suggest the important role of PIP_2_ in hAE2 substrates exchange process. Sequence alignments showed that residues contributing to the PIP_2_ binding site are conserved among AE2 homologs and basic residues for PIP_2_ binding are also highly conserved among most human SLC4 family members (Fig. 3i), implying the possibility of a shared PIP_2_ regulatory function among them. The activity attenuating effects of the K1147 and H1148 mutations of hAE2 is consistent with the corresponding K835 mutation in hAE1 and H1144 mutation in mAE2^38–40^. The H1144 mutation in mAE2 also changed the pH sensitivity of AE2-mediated substrate transport, which suggests that PIP_2_ might participate in the pH regulation of AE2 activity^38,40^.

### TMD of AE2 in complex with PIP_2_

**Fig. 4:**
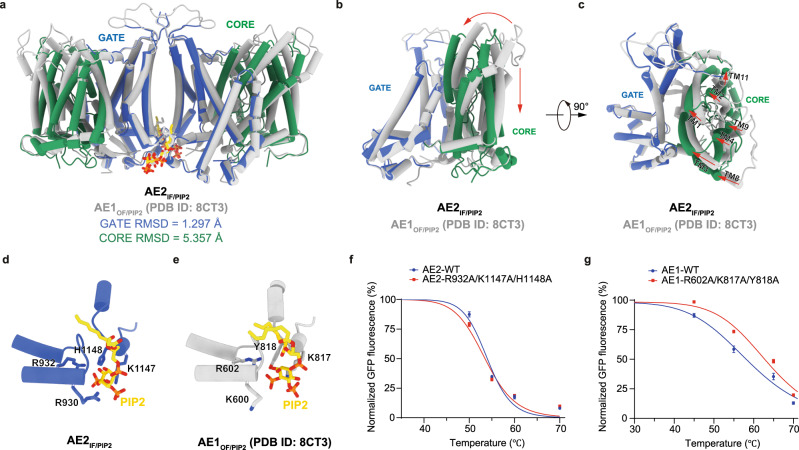
TMD of AE2 in complex with PIP_2_. **a** Structural comparison of the PIP_2_-bound TMDs of inward-facing AE2_IF/PIP2_ and outward-facing AE1_OF/PIP2_ (8CT3) aligned by the gate domain. The gate domain and core domain of AE2 are colored in blue and green, respectively. AE1_OF/PIP2_ is colored in gray. **b**, **c** Structural comparison of one protomer of the AE1_OF/PIP2_ TMD (gray) and the AE2_IF/PIP2_ TMD (colored) viewed from the side (**b**) and from the extracellular side (**c**). Displacements of the core domain from outward-facing AE1_OF/PIP2_ to inward-facing AE2_IF/PIP2_ are indicated by red arrows. **d**, **e** Comparison of the PIP_2_ binding site of AE2_IF/PIP2_ (**d**) and AE1_OF/PIP2_ (**e**). PIP_2_, residues formed the PIP_2_ binding site of AE2_IF/PIP2_ and the corresponding residues in AE1_OF/PIP2_ are shown as sticks. **f** Thermal shift stabilization of mCherry-tagged wild-type AE2 (blue) and AE2 with mutations in the PIP_2_ binding sites (red). The mCherry fluorescence of AE2 dimer peak after heating was normalized to that without heating. The data are presented as mean values ± SD. The number of independent experiments for both wild-type AE2 and triple mutant AE2-R932A/K1174A/H1148A is 3. The Tm_50_ values for wild-type AE2 and triple mutant AE2 are 54.0 °C and 53.5 °C, respectively. The apparent melting temperature Tm was calculated using a sigmoidal four-parameter logistic regression function and a *p* value of 0.24 (not significant) was determined by a two-sided F-test. **g** Thermal shift stabilization of wild-type AE1 (blue) and AE1 with disrupted PIP_2_ binding sites (red). The data are normalized fluorescence and presented as mean values ± SD as in **f** (*n* = 3 independent experiments for both wild-type and triple mutant AE1). The Tm_50_ values for wild-type AE1 and AE1-R602A/K817A/Y818A are 57.2 °C and 60.4 °C, respectively. The apparent melting temperature Tm was calculated using a sigmoidal four-parameter logistic regression function and a *p* value of 2.15× 10^−7^ (<0.05; significant) was determined by a two-sided F-test.

Recently solved structures of AE1 in complex with protein 4.2 and ankyrin were all in the outward-facing conformation^22,23^. Cryo-EM densities reported by Vallese et al. showed that PIP_2_ sits in the middle of the AE1 TMD dimer interface and interacts with AE1 through K600, R602, and R603 of one protomer and K817 of the other protomer^23^. Structural comparison of the TMDs of AE1 in the outward-facing state (PDB ID: 8CT3, referred to as AE1_OF/PIP2_) and AE2_IF/PIP2_ aligned by their gate domains showed that PIP_2_ binds at the similar position of the dimer interface (Fig. 4a). The arrangement of TM and intracellular helices of AE1_OF/PIP2_ gate domain is similar to that of AE2_IF/PIP2_ with a RMSD of 1.297 Å (Fig. 4a). The core domain of AE2_IF/PIP2_ rotates towards the gate domain and shifts downward toward the cytosolic side relative to core domain of AE1_OF/PIP2_ with a RMSD of 5.357 Å (Fig. 4a–c). The ‘pore’ region formed by TM1, TM3, TM8 and TM10 undergoes a 15.5 ° rotation and a centroid consisting of G769 and T1058 (G466 and T728 in AE1) shifts 6.4 Å from the outward-facing state to the inward-facing state (Supplementary Fig. 4a). TM arrangements within the core domain of AE1_OF/PIP2_ and AE2_IF/PIP2_ showed little difference during structure transition (RMSD = 1.009 Å) (Supplementary Fig. 4b). The conformation of AE2_IF/PIP2_ is similar to that of AE2_inter_^basic-KNO3^ as determined by Zhang et al.^25^ with a gate domain RMSD of 1.368 Å and a core domain RMSD of 1.707 Å when aligned by the gate domain (Supplementary Fig. 4c). Alignment of the TMDs of AE2_IF/PIP2_ and AE2^acidic-KNO3^ showed that the intracellular side of TM7 shifts toward the core domain, and inward rotational displacements of the core domains of AE2^acidic-KNO3^ are greater than that of AE2_IF/PIP2_ with a RMSD of 2.32 Å and a shift of centroids of 1.8 Å (Supplementary Fig. 4d, e).

Mutagenesis analysis of the PIP_2_ binding site showed that interaction between PIP_2_ and the AE2 gate domain is important for the substrate permeation process as introduction of the R932A, K1147A and H1148A mutations significantly reduced AE2 activity (Fig. 3g). To test whether PIP_2_ facilitates substrate permeation by stabilizing the dimer interface through electrostatic interactions (Fig. 4d, e), we measured the thermostability of wild-type AE2 and AE2 with the R932A, K1147A and H1148A mutations and the thermostability of AE1 and AE2 at different pH (Fig. 4f, g and Supplementary Fig. 4f–h). The results indicated that AE2 is more sensitive to pH than AE1 (Supplementary Fig. 4f–h) and the thermostability of AE2 is not affected by these mutations (Fig. 4f). The corresponding mutations R602A, K817A and Y818A in AE1 increased its thermostability (Tm_50_ of wild-type and R602A/K817A/Y818A mutant AE1 are 57.2 °C and 60.4 °C, respectively) suggesting these mutations stabilize AE1 by rigidifying its conformation (Fig. 4g). These observations suggest that PIP_2_ did not facilitate the AE2 substrate permeation process by stabilizing TMD dimerization, but might regulate AE2 activity through a different mechanism, such as allosterically interfering with the interaction between the NTD and the TMD or regulating conformational changes of the TMD.

### Interactions between NTD and TMD of AE2 in absence of PIP_2_

**Fig. 5:**
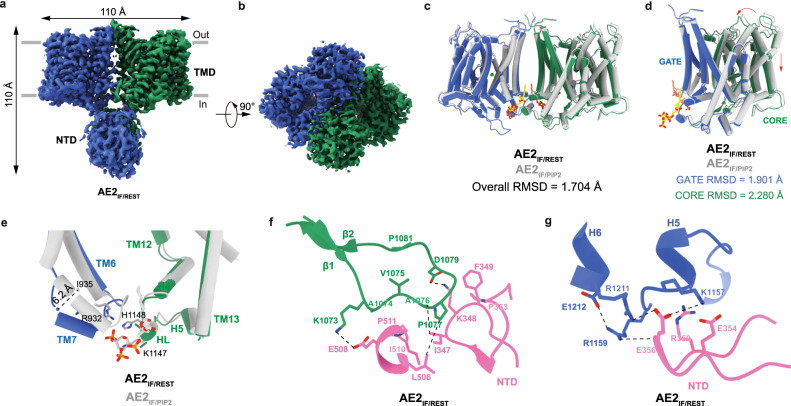
Structure of AE2 in the absence of PIP_2_. **a**, **b** Cryo-EM density map of AE2_IF/REST_ state (AE2 with R932A/K1147A/H1148A mutations in the inward-facing conformation), viewed from the side (**a**) and the cytoplasmic side (**b**). The approximate position of the lipid bilayer is indicated by gray bars. The two AE2 protomers are colored in green and blue, respectively. **c** Structural comparison of the TMDs of AE2_IF/REST_ and AE2_IF/PIP2_ aligned by the gate domain. Two PIP_2_ molecules and the TMD of AE2_IF/PIP2_ are colored in yellow, orange and gray, respectively. The two protomers of AE2_IF/REST_ are colored in blue and green, respectively. **d** Structural comparison of one protomer of each of the AE2_IF/PIP2_ and AE2_IF/REST_ states. The gate domain and core domain of AE2_IF/REST_ are colored in blue and green, respectively. Two PIP_2_ molecules and the TMD of AE2_IF/PIP2_ are colored in yellow, orange and gray, respectively. Arrows indicate the displacements of the core domain from the AE2_IF/PIP2_ state to the AE2_IF/REST_ state. **e** Structural comparison of the PIP_2_ binding site in the AE2_IF/PIP2_ and AE2_IF/REST_ states. The Cα distance between I935 in the AE2_IF/PIP2_ and AE2_IF/REST_ states are shown as a dashed line. The gate domain and core domain of AE2_IF/REST_ are colored in blue and green, respectively. AE2_IF/PIP2_ is colored in gray. **f** Interface of the AE2_IF/REST_ NTD and the core domain of the TMD. The loop connecting TM10 to TM11 is colored in green and the NTD is colored in pink. Residues within the interface are shown as sticks and interactions between residues are indicated by dashed lines. **g** Interface of AE2_IF/REST_ NTD and gate domain of TMD of the other protomer. NTD is colored in pink and gate domain of the other protomer is colored in blue. Residues within the interface are shown as sticks and interactions between residues are indicated by dashed lines.

To investigate whether PIP_2_ regulates AE2 substrate exchange activity by allosterically disrupting the interface of the AE2 TMD and NTD, we introduced mutations (i.e., R932A, K1147A, and H1148A) into AE2 and then determined their structures. The mutant AE2 proteins were expressed and purified under the same conditions as wild-type AE2 and a map was reconstructed at an overall resolution of 3.3 Å (Supplementary Fig. 5). Analyses showed a strong density of the AE2 NTD which was not identified in the AE2_IF/PIP2_ state (Fig. 5a, b). The mutant AE2 TMD also adopted an inward-facing conformation with a RMSD of 1.704 Å compared with the TMD of AE2_IF/PIP2_ state (Fig. 5c). Because the substrate exchange activity of AE2 harboring the R932A/K1147A/H1148A mutations was significantly reduced, we designate this structure as the AE2_IF/REST_ state. Aligning the TMD of the AE2_IF/REST_ state with that of the AE2_IF/PIP2_ state by gate domain showed that inward rotational displacement of the AE2_IF/REST_ core domain is greater (with a RMSD of 2.280 Å) than the displacement of core domain in the AE2_IF/PIP2_ state (Fig. 5c, d and Supplementary Fig. 6a). PIP_2_ densities were absent from the dimer interface of the AE2_IF/REST_ gate domain (Fig. 5a, b). Without the binding of PIP_2_, the intracellular half of TM7 shifted 6.2 Å (distance between Cα of I935) toward the core domain and the loop connecting TM6 to TM7 moved upward away from PIP_2_ (Fig. 5e). Overall, the conformation of AE2 with mutations in the PIP_2_ binding site is similar to the AE2 conformation at acidic pH (AE2^acidic-KNO3^, PDB ID: 8GVH) with a 1.4 Å displacement of the substrate binding site, suggesting the regulatory mechanism of PIP_2_ is similar to that of acidic intracellular pH (Supplementary Fig. 6b, c). Interfaces between TMD and NTD in the AE2_IF/REST_ state are also consistent with the AE2^acidic-KNO3^ state (Supplementary Fig. 6d, e).

Loops and β-sheets between TM10 and TM11 of AE2_IF/REST_ state (missing in the AE2_IF/PIP2_ state due to their flexibility) stabilized the interface between the NTD and the TMD through several electrostatic interactions (between E508 and K1073, K348, and D1079) and hydrophobic interactions (between I347, F349, P363, L506, I510, P511, A1074, V1075, A1076 and P1077) (Fig. 5f). Electrostatic interactions formed between E356 in the NTD, and K1157 and R1211 on the other protomer further stabilized the interaction between the AE2_IF/REST_ TMD and NTD (Fig. 5g). Ankyrin complexes solved by others display extensive interfaces between AE1 and protein 4.2 suggesting the involvement of protein 4.2 in the regulation of AE1^22,23^. Thus, interaction between AE1 and protein 4.2 results in a rev-V shape of the AE1 NTD and hinders direct contact of its TMD and NTD^22^. Vallese et al.^23^ suggested that the interaction of protein 4.2 with the AE1 TMD was at least partially mediated by PIP_2_ and R126 and K127 of protein 4.2^23^. Therefore, the NTD of AE1 might adopt a V shape conformation without the binding of protein 4.2. At the same time, the loss of PIP_2_ at the dimer interface further reinforced the TMD-NTD interaction and locked AE1 in an inward-facing conformation. This interpretation is consistent with a recently solved structure of AE1 which showed that its NTD is less flexible in the inward-facing conformation^24^. Thus, the mechanism of PIP_2_-mediated regulation of AE2 activity may be universal in the AE subfamily proteins and may be involved even more generally in the regulation of all the SLC4 family transporters as previously proposed^28,31,32^.

## Discussion

**Fig. 6:**
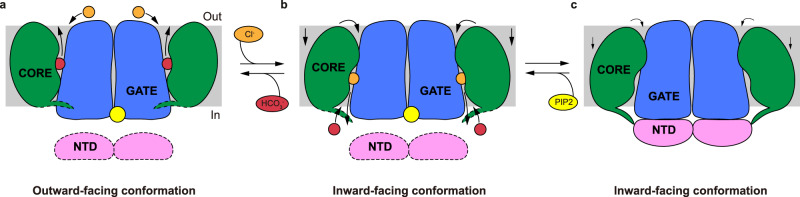
Model for AE2 conformational changes regulated by PIP_2_. **a**–**c** Cartoon model of the side view of AE2. The gate domain, core domain, NTD, PIP_2_, Cl^−^, and HCO_3_^−^ are colored in blue, green, pink, yellow, orange, and red, respectively. The tail of the core domain represents the intracellular loop connecting TM10 to TM11. The loop between TM10 and TM11 and the NTD in **a** and **b** is outlined with dashed lines because its flexibility precludes its detection in cryo-EM maps. In the presence of PIP_2_, HCO_3_^−^ is released to the extracellular side of the outward-facing conformation of AE2 (**a**) and Cl^−^ is released to the intracellular side when in its inward-facing conformation (**b**). In the absence of PIP_2_, the NTDs interact with TMDs and lock AE2 in an inward-facing resting state (**c**).

PIP_2_ is a phospholipid found in the inner leaflet of plasma membranes and is required for the function of many ion channels and ion transporters. PIP_2_ dependency has been previously studied in Kir6.x channels, TRP channels, KCNQ channels, and ion transporters including several SLC4 transporter family proteins^41^. Previous studies showed that the mobility and activity of AE1 are regulated by lipids, such as cholesterol, and molecular dynamics simulations showed that PIP_2_ on the inner leaflet of the bilayer prefers to interact with AE1. This suggested the involvement of PIP_2_ in the regulation of activity and complex formation of AE1^42–45^. Previous studies showed that PIP_2_ stimulates NBCe1 (SLC4A4) activity through a dual pathway^31^. Moreover, recently solved structures of AE1 identified the binding sites of PIP_2_ molecules and the authors proposed that PIP_2_ may participate in the formation of ankyrin-1 complexes^23^. Our structural and functional studies described here reveal that PIP_2_ disrupts the interaction between the TMD and NTD of hAE2, preventing the inhibitory effect of the NTD on conformational transitions of AE2 and thus promotes substrate transport (Fig. 6). The positively charged PIP_2_ binding pocket of AE2 locates to a similar position of the outward-facing state AE1 at the dimer interface of the TMDs (Figs. 2a and 4a). Although PIP_2_ binds at the AE2 TMD dimer interface, disruption of its binding site by mutating R932, K1147 or H1148 had a relatively mild impact on its structural stability and plasma membrane trafficking comparing with its effect on its substrate exchange activity (Fig. 4f and Supplementary Fig. 3k). Mutagenesis analysis indicated the interaction between lipid and AE2 is mainly attributable to R932, K1147 and H1148, and is involved in the regulation of transport activity (Fig. 3a, b). Residues of AE2 forming the lipid binding pocket are highly conserved, indicating that PIP_2_ might regulate the activity of other AE subfamily proteins, and perhaps even extended SLC4 family members, in a similar manner (Fig. 3i). The TMD of AE proteins may adopt either an inward-facing state or an outward-facing state in the presence of PIP_2_ to carry out the substrate exchange process (Figs. 4a and 6a, b), which is supported by the presence of PIP_2_ in cryo-EM densities of outward-facing AE2_out_^basic-KNO3^ (PDB ID:8GVA) and inward-facing AE2^NaCl^ (PDB ID:8GV9) as determined by Zhang et al. (Supplementary Fig. 6g, f)^25^. When the binding pocket of PIP_2_ was conformationally altered by charge-neutralizing mutations or by acidic intracellular pH, the intracellular side of TM7 shifted toward TM1 of the core domain through hydrophobic interactions (Fig. 5e, f). This observation is consistent with the conformation changes reported for AE2^NaCl^ and AE2^acidic-KNO3^ (PDB ID:8GVH). The NTD further stabilizes this conformation by interacting with the TMD (Fig. 5f, g). The structure of AE2_IF/REST_ suggests that PIP_2_ activates AE2 activity by allosterically interfering with both the interaction between TM7 and TM1, and the binding of the NTD to the TMD (Fig. 6c).

The regulation of AE2 by pH has been extensively studied^13,40,46–48^. Mutations of H1144 of mAE2, corresponding to H1148 of hAE2, lead to a decrease in substrate exchange activity and pH sensitivity^40^. Based on the facts that positively charged residues form the lipid binding site, PIP_2_ is protonated at acidic pH and electrostatic interactions stabilized TMD and NTD interface, these give rise to a hypothesis that PIP_2_ may function as a pH sensor to orchestrate the conformational changes of NTD and TMD regulated by environmental pH. This hypothesis is supported by the features of currently solved structures of AE2 under acidic and alkaline pH^25^. Thus, PIP_2_ binds to the TMDs of AE2 at alkaline pH and the TMDs adopt an inward-facing or outward-facing conformation according to the binding substrates (Supplementary Fig. 6f and Fig. 6a, b). The flexibility of the NTD under substrate transport conditions is consistent with previous studies that showed that removal of the NTD had no obvious effect on AE2 activity^48^. Thus, PIP_2_ functions as an activator and the C-terminal loop acts as an inhibitor of AE2. Without the binding of PIP_2_, TM7 shifts toward the core domain and stabilizes the interaction between the TMD and NTD. Acidic intracellular pH induces the insertion of the C-terminal loop into the cavity between the core and gate domains, which further stabilizes the inward-facing resting state by preventing substrate binding^25^. This proposed regulatory mechanism is supported by previous functional studies and can explain the pivotal role of PIP_2_ and the NTD in pH sensing, conformation changes, and the substrate exchange process of AE2^18,46^.

In summary, our structural and functional analysis of AE2 in the presence and absence of PIP_2_ propose PIP_2_ as a regulatory ligand of AE2 and provides structural insights into the regulatory mechanism of conformational transitions and substrate transport activity of this transporter.

## Methods

### Cell lines

FreeStyle 293 F (Thermo Fisher Scientific) suspension cells were cultured in 293 Expression Medium (Gibco) supplemented with 1% fetal bovine serum (FBS) at 37 °C, with 6% CO_2_ and 70% humidity. Sf9 insect cells (Thermo Fisher Scientific) were cultured in Sf-900 III SFM medium (Thermo Fisher Scientific) at 27 °C. HEK293T cells (ATCC) were cultured in DMEM basic (Thermo Fisher Scientific) supplemented with 10% FBS at 37 °C, with 6% CO_2_ and 70% humidity.

### Expression constructs

Human *Ae2* (isoform a, UniProtKB accession: P04920-1) was cloned into the C-terminal GFP tagged BacMam expression vector^49^, which also contains C-terminal His tag. For functional studies, AE2 was cloned into a modified C-terminal GFP-tagged BacMam or mCherry-tagged pCCL expression vector^50^. To facilitate the measurement of plasma membrane expression of various AE2 constructs, a HA-tag (YPYDVPDYA) was inserted between Thr870 and Try871 of *SLC4A2* cloned into the BacMam vector.

### Expression and purification of AE2

AE2 proteins were expressed using the BacMam system as described previously with minor modifications^49,50^. Briefly, cells were harvested 48 h after infection with baculovirus and membrane pellets were extracted. For purification, membrane pellets were homogenized in buffer containing 20 mM HEPES at pH 7.4 and 150 mM NaCl (HBS) and then solubilized in HBS with 1% LMNG (Anatrace) and 0.1% CHS (Anatrace). Unsolubilized material was removed by centrifugation at 98,000 g for 30 min. The supernatant was supplemented with 20 mM imidazole and loaded onto a 5 ml Ni-NTA column (GE Healthcare). The beads were first washed with HBS containing 50 μM GDN (Anatrace) and then by HBS with 50 μM GDN and 20 mM imidazole. Protein was eluted using HBS with 50 μM GDN and 250 mM imidazole, and tags were removed by TEV protease. Proteins were concentrated using a 30-kDa cut-off concentrator (Sartorius) and loaded onto Superose 6 increase 10/300 GL column (GE Healthcare) running in HBS with 50 μM GDN. The peak corresponding to hAE2 was collected for cryo-EM sample preparation.

### Cryo-EM sample preparation and data acquisition

Cryo-EM samples were concentrated in a buffer containing 50 μM GDN, 150 mM NaCl, and 20 mM HEPES at pH 7.4 and loaded onto glow-discharged Quantifoil 1.2/1.3 gold grids and frozen as described previously^49^. Cryo-grids were screened in Titan Krios microscope (Thermo Fisher Scientific) at 300 kV for data acquisition. Images were collected using a K2 camera (Gatan) mounted post a quantum energy filter with a 20 eV slit and operated under super-resolution mode with a pixel size of 0.821 Å at the object plane. Defocus values were set to range from −1.8 μm to −2.0 μm for data collection. Data were acquired using Serial-EM-3.6.11. The dose rate on the detector was 12.0 e^−^s^−1^A^−2^ and the total exposure was 52 e^−^A^−2^. Each 4.4 s movie was dose-fractionated into 40 frames.

### Image processing

CryoSPARC software-3.1.0 was used for structural analysis^51^. The original movies were gain-corrected, motion-corrected and binned in a Patch motion correction step. The summed micrographs were dose-weighted with a pixel size of 0.821 Å. The defocus values of corrected micrographs were estimated with Patch CTF (Contrast Transfer Function) estimation in CryoSPARC. Auto-picking was done using Blob Picker in CryoSPARC. Auto-picked particles were extracted from dose-weighted micrographs by a binning factor of 2. Datasets were subjected to 2D classification using CryoSPARC. Particles of AE2_IF/PIP2_ yielding from 2D classification were subjected to ab initial model generation with *n* = 3 using cryoSPARC-3.1.0^51^ and further refined against the initial model using non-uniform and local refinement in cryoSPARC-3.1.0 to reach a resolution of 3.17 Å. Particles of AE2_IF/REST_ yielding from 2D classification were similarly subjected to ab initial model generation with *n* = 2 using cryoSPARC. Particles from good classes were used as reference for Topaz Train and auto-picking was done by Topaz Extract^52^. Particles were screened as described above and refined against an initial model using non-uniform refinement and local refinement in cryoSPARC-3.1.0 to reach a resolution of 3.26 Å.

### Model building

We used alphafold^53^ to build initial models of the NTD and TMD of hAE2 and merged them in Coot^54^ and docked into the cryo-EM map with UCSF Chimera-1.14^55^. Models were manually rebuilt in Coot-0.9.2^56^ and further refined by Phenix1.19.2-4158^57^. The residues contained in the final models are indicated in Supplementary Table 1. Figures were prepared using Pymol-1.7.0.5 (Schrodinger, LLC.) and UCSF ChimeraX-1.5^56^.

### Flow cytometry

Flow cytometry was used to assess the plasma membrane expression of WT and mutant AE2. 293 F cells were transfected with HA-tagged AE2 constructs carrying a C-terminal GFP tag. Cells were collected 24 h post-transfection and incubated with HA tag antibody (PE conjugated, BioLegend, cat# 901518, clone 16B12, FC/ICFC) for 30 min at room temperature, followed by acquisition on a flow cytometer (BD LSRFortessa^TM^) and data were analyzed using FlowJo v10.6.2 software. The expression level was calculated as (MFI_Q2, PE_ × Cell number_Q2_)/ (MFI_Q2, FITC_ × Cell number_Q2_ + MFI_Q3, FITC_ × Cell number_Q3_), in which MFI means mean fluorescent intensity and Qn indicates specific gate as shown in supplementary figures.

### Anion exchange activity assays

HEK293T cells were transfected with C-terminally mCherry-tagged wild-type and mutant hAE2. Cells were seeded onto glass coverslips (Ø 42 mm) 24 h after transfection. 12 h later, 5 μM BCECF-AM (ThermoFisher Scientific, Invitrogen^TM^, USA) was added to the cells at 37 °C, 5% CO_2_ for 30 min. Then the coverslips were mounted in a POC-R2 Cell Cultivation System (open perfusion, flat version, PeCon®). The HEK293T cells were initially bathed in ${{{\mbox{Cl}}}}^{-}$-free Ringer’s buffer containing 140 mM sodium gluconate, 5 mM potassium gluconate, 1 mM calcium gluconate, 5 mM glucose, 1 mM MgSO_4_, 2.5 mM NaH_2_PO_4_, 25 mM NaHCO_3_, 30 μM Ethylisopropylamiloride and 10 mM HEPES at pH 7.4. After cells equilibrated reached balanced states, the bath solution was replaced with ${{{\mbox{Cl}}}}^{-}$-containing Ringer’s buffer as above but containing 140 mM NaCl instead of 140 mM sodium gluconate. Intracellular fluorescence was then recorded every 10 s (excitation ratio 488 nm/405 nm; emission 530 nm) and calibrated at the end of each experiment by the addition of a mixture of 10 μM valinomycin and 10 μM nigericin (both Sigma-Aldrich). ${{{\mbox{Cl}}}}^{-}$-driven base flux was calculated as d[H_in_^+^]/dt × β_Total_ where β_Total_ is the total pH buffering capacity, and consist of β_intrinsic_ and β_HCO3_^−^, where β_intrinsic_ was calculated by addition of NH_4_Cl after cells reached steady state in ${{{\mbox{Cl}}}}^{-}$-containing Ringer’s buffer^58^), and d[H_in_^+^]/dt was measured using linear curve fitting after increasing bath ${{{\mbox{Cl}}}}^{-}$- from zero to 140 mM at pHi 7.2. 1 μM wortmannin (MedChemExpress, cat# HY-10197) or 30 μM m-3m3FBS (MedChemExpress, cat# HY-19619) was added to cells along with BCECF-AM. Fluorescence was recorded using an LSM880 instrument (Carl Zeiss) and analyzed using its supporting software.

### Thermal stability assays

HEK293F cells were transfected with AE expressing constructs carrying a C-terminal mCherry tag. Cells were collected 40 h post-transfection and solubilized in buffer containing 150 mM NaCl, 1% LMNG, 0.1% CHS and 20 mM HEPES at pH 7.4. Cell lysates was centrifuged at 98,000×*g* for 30 min and supernatants were divided into several aliquots. Aliquots were heated at different temperatures for 15 min. After centrifuging at 98,000×*g* for 30 min, supernatants were loaded onto a Superose 6 increase column (GE Healthcare) for fluorescence size exclusion chromatography analysis^59^. The remaining dimeric AEs peak height of the heated sample was divided by the peak height of the non-heated sample to calculate relative thermal stability. The assays were independently repeated 3 times.

### Quantification and statistical analysis

Global resolution estimations of cryo-EM density maps are based on the 0.143 Fourier Shell Correlation criterion^60^. The local resolution map was calculated using cryoSPARC-3.1.0^51^. IBM SPSS Statistics v27.0.0.0 and Prism GraphPad software v9.3.0 was used for statistical analysis. The number of biological replicates (N) and the relevant statistical parameters for each experiment (such as means or SD) are described in the figure legends. No statistical methods were used to pre-determine sample sizes.

### Reporting summary

Further information on research design is available in the Nature Portfolio Reporting Summary linked to this article.

### Supplementary information

Supplementary Information

Peer Review File

Reporting Summary

### Source data

Source Data

## Data Availability

The data that support this study are available from the corresponding authors upon request. Cryo-EM maps and atomic coordinates are deposited in the Electron Microscopy Data Bank (EMDB) and Protein Data Bank (PDB): EMD-36448 (AE2_IF/PIP2_) and PDB: 8JNI (AE2_IF/PIP2_); AE2_IF/REST_: EMD-36449 (AE2_IF/REST_) and PDB: 8JNJ (AE2_IF/REST_). 8CT3, 8GVA, 8GVH, EMD-26143, EMD-34292 and EMD-34288 are already available on Protein Data Bank. The source data underlying Figs. 3b–h, j and 4f, g, and Supplementary Figs. 3k and 4h are provided as a Source Data file. Reagents generated in this study will be made available freely for academic research purposes and/or may require a Materials Transfer Agreement. Source data are provided with this paper.
