# Supplementary material for: Structural and functional insights into the lipid regulation of human anion exchanger 2
Source: Nat Commun. 2024 Jan 26;15:759. doi: 10.1038/s41467-024-44966-0 (PMC10810954; doi:10.1038/s41467-024-44966-0)
Supplement: Supplementary file 1 — Supplementary Information [file 41467_2024_44966_MOESM1_ESM.pdf]

Supplementary information for

Structural and functional insights into the lipid regulation of human anion exchanger 2

Weiqi Zhang, Dian Ding, Yishuo Lu, Hongyi Chen, Peijun Jiang, Peng Zuo, Guangxi Wang, Juan Luo, Yue Yin, Jianyuan Luo and Yuxin Yin*

*To whom correspondence should be addressed: Yuxin Yin (yinyuxin@hsc.pku.edu.cn)

This file includes:
Supplementary Figs. 1-6
Supplementary Table 1

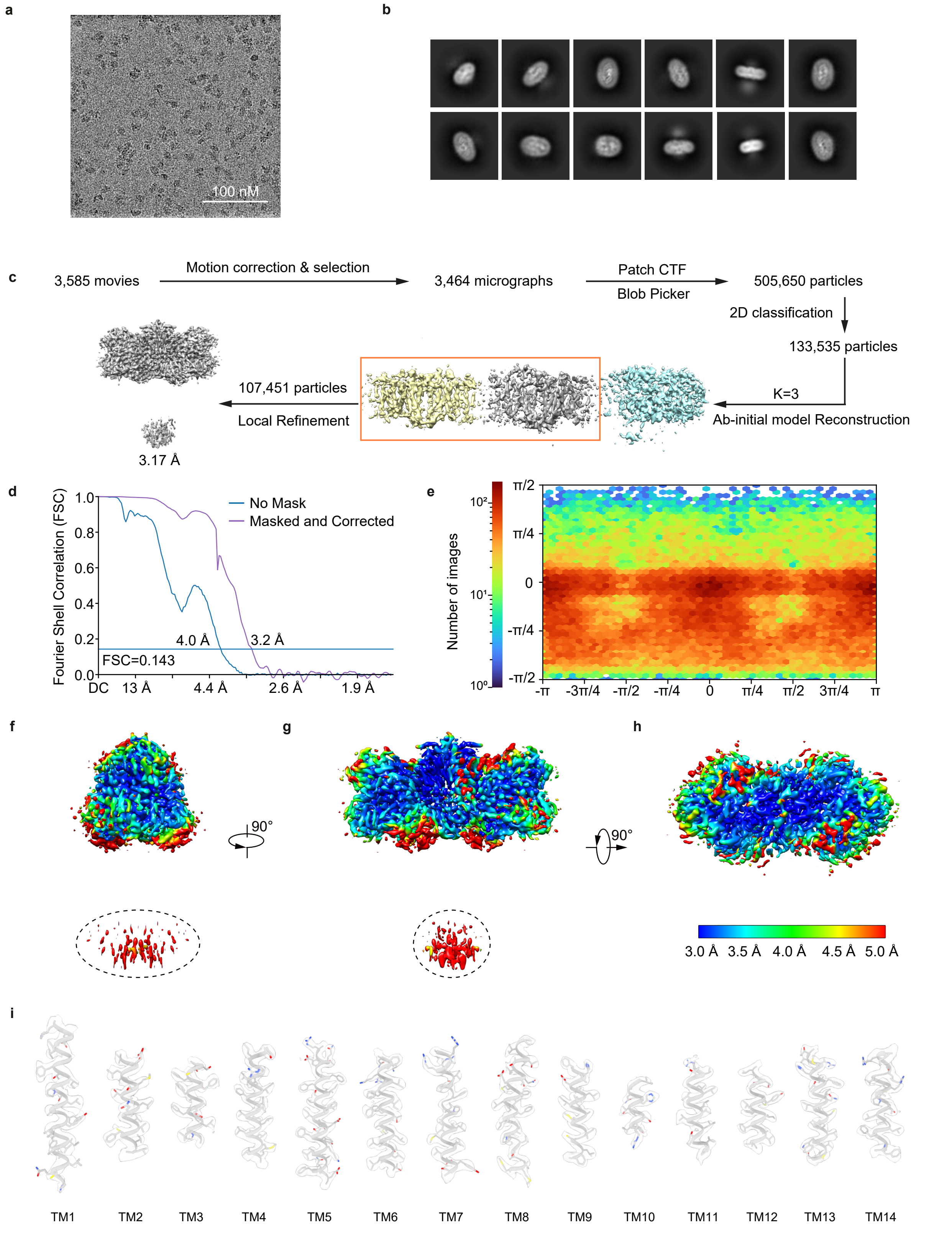

Supplementary Figure. 1: Workflow for cryo-EM data processing of AE2$_{IF/PIP2}$.
a Representative image from a dataset consisting of 3,464 motion-corrected micrographs.
b Two-dimensional class averages of the sample.
c EM data processing workflow. Three classes generated by ab-initial reconstruction are colored in yellow, grey and blue, respectively. Particles in the yellow and grey classes enclosed in the orange box were used for finial reconstruction.
d Resolution estimation of the AE2 map, based on the criterion of the FSC 0.143 cut-off.
e Angular distribution of the final reconstruction.
f-h Local resolution maps in side views (**f** and **g**) and top view (**h**) colored according to the bar in the lower right of the figure. Densities indicated by the dashed circle may belong to the flexible NTD.
i Cryo-EM densities of the transmembrane helices of the AE$_{IF/PIP2}$ state, superimposed with the corresponding stick models.

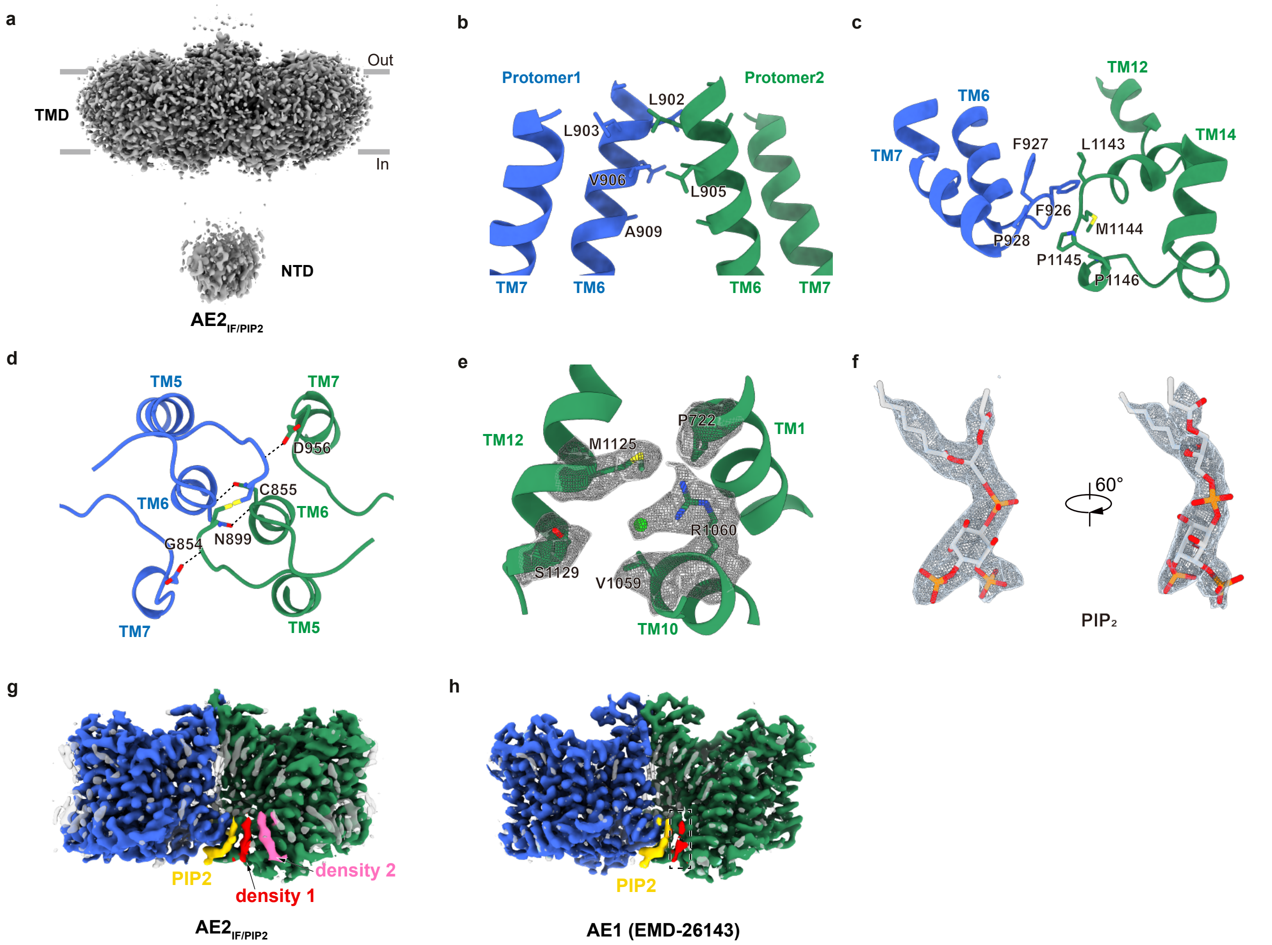

Supplementary Figure. 2: Structural feature of AE2$_{IF/PIP2}$.

a Sharpened map of AE2$_{IF/PIP2}$ contoured at 0.11 level (4.7 σ).

b Hydrophobic interactions of the dimer interface as viewed from the extracellular side. The two protomers of AE2 are colored in blue and green, respectively. Residues within the interface are shown as sticks.

c Hydrophobic interactions of the AE2 dimer interface viewed from the cytoplasmic side. The two protomers of AE2 are colored in blue and green, respectively. Residues within the interface are shown as sticks.

d Interaction of extracellular loop of TMD. Two protomers are colored in blue and green, respectively. Interacting residues (G854-D956, N899-N899, C855-C855) are shown as sticks and interactions are indicated by dotted lines.

e Close-up view of the substrate binding site of AE2$_{IF/PIP2}$. Densities of Cl^- and surrounding residues are shown as mesh.

f Cryo-EM density of PIP_2 in the AE2$_{IF/PIP2}$ state.

g Map of AE2$_{IF/PIP2}$ state. The two protomers, PIP_2, densities corresponding to cholesterol in 8CT3 (density 1) and CHS in 8GVH (density 2) are colored in blue, green, yellow, red and pink, respectively.

h Map of AE1(EMD-26143). The two protomers, PIP_2 and densities corresponding to cholesterol in 8CT3 (indicated by dashed line) are colored in blue, green, yellow and red, respectively.

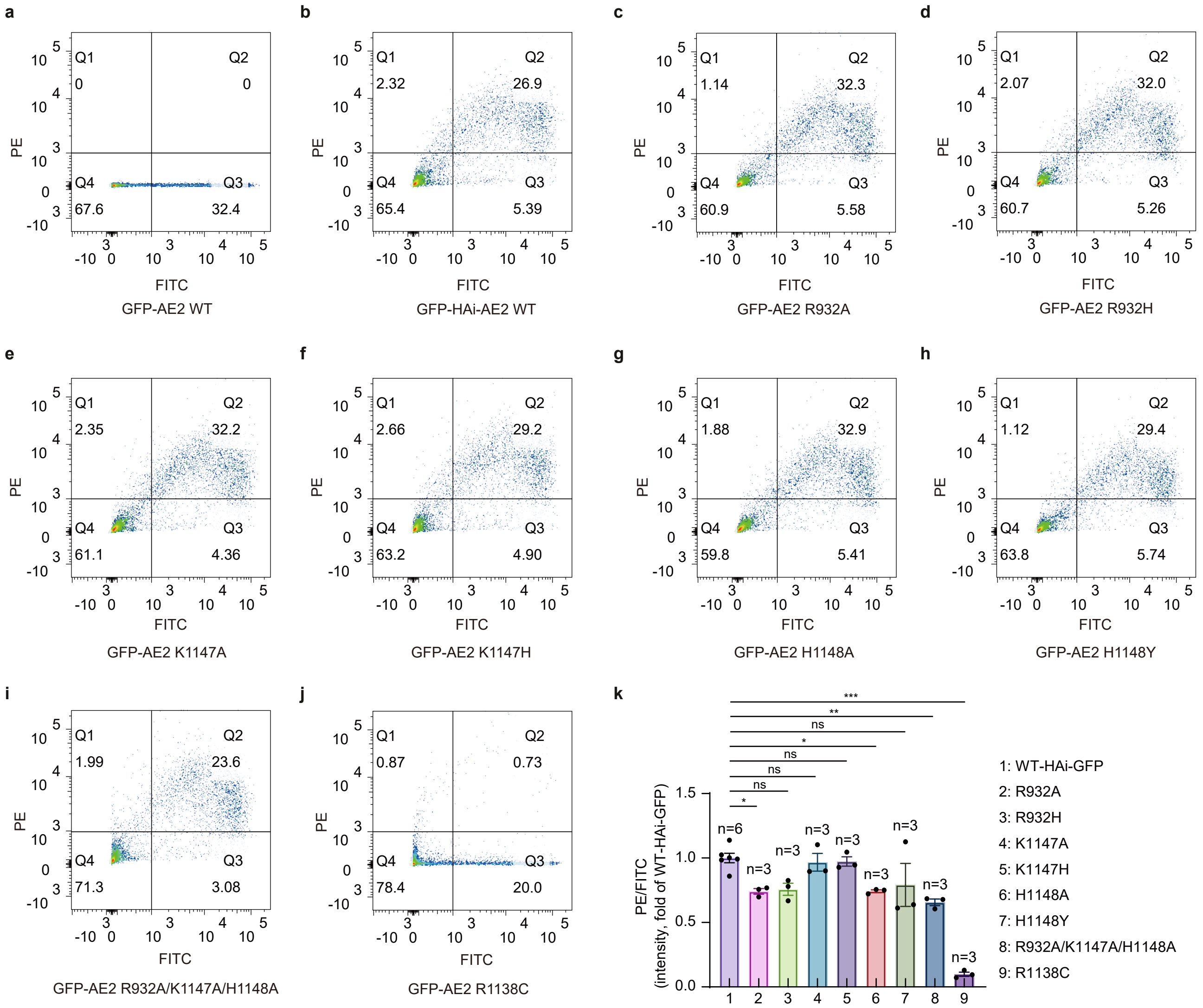

Supplementary Figure. 3: Membrane expression of wild-type and mutant HA-tagged AE2.

a-j Example of flow cytometry cell clustering diagrams of cells transfected with AE2 constructs containing an extracellular HA-tag or not. The horizontal and vertical coordinates represent the intensity of GFP fluorescence (FITC signal) and HA-tag (PE signal), respectively.

k Plasma membrane expression levels of wild-type and mutant HA-tagged AE2. PE/FITC intensities (anti-HA antibody/GFP fluorescence) of the mutant AE2 are normalized to wild-type AE2. Data of WT-HAi-GFP (n=6 biologically independent experiments), R932A (n=3, p=0.01), R932H (n=3, p=0.14), K1147A (n=3, p=1.00), K1147H (n=3, p=1.00), H1148A (n=3, p=0.01), K1148Y (n=3, p=0.96), R932A/K1147A/H1148A (n=3, p=0.0026) and R1138C (n=3, p=4.00 × 10^{-6}) are shown as mean values ± SD of n ≥ 3 biologically independent experiments (p values were calculated by one-way ANOVA and post hoc Dunnett's test, *p < 0.05, **p < 0.01, ***p < 0.001).

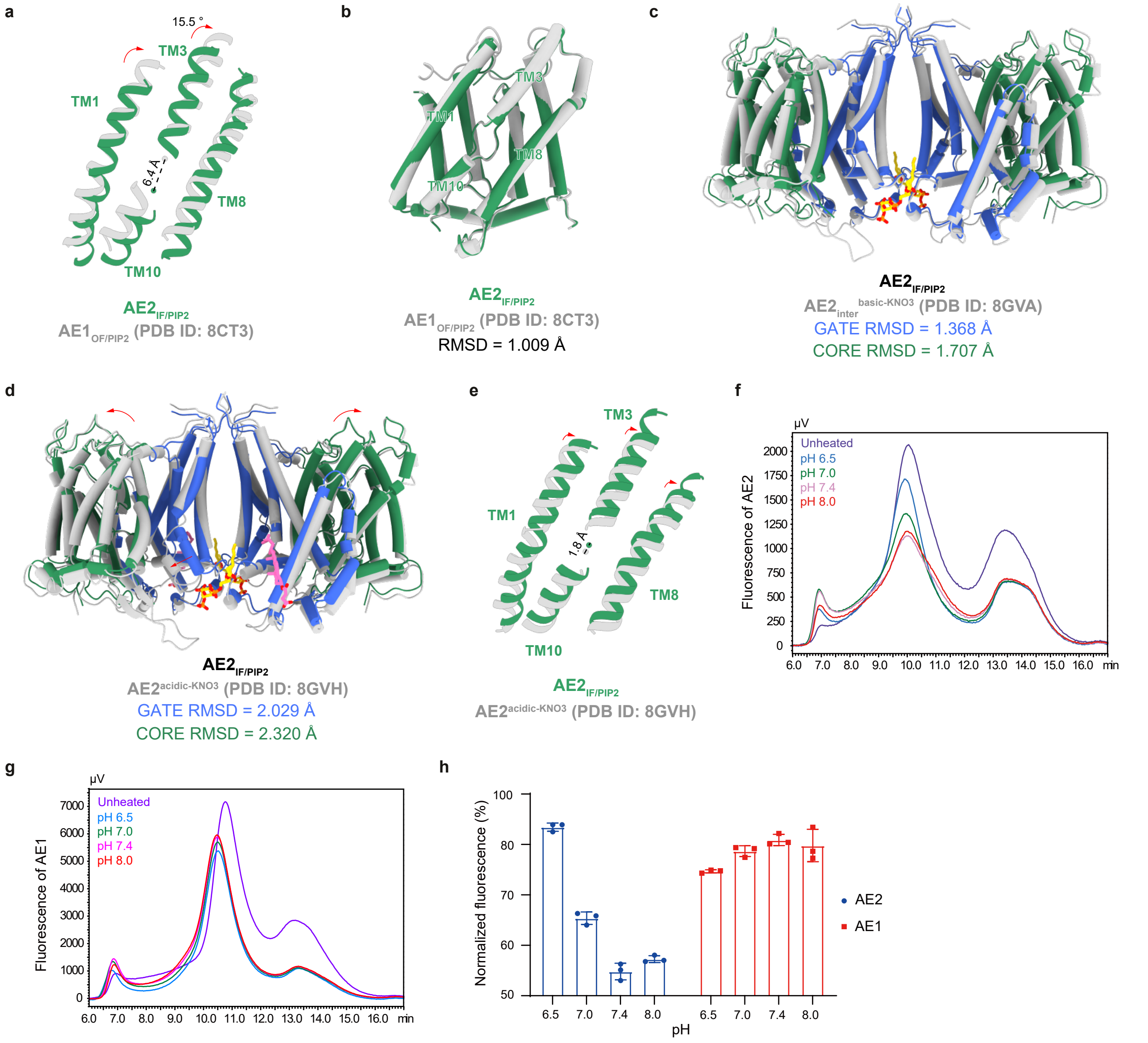

Supplementary Figure. 4: Comparison of AE2$_{IF/PIP2}$ with AEs in different states.

a Structural comparison of the substrate coordination site of AE2$_{IF/PIP2}$ and AE1$_{OF/PIP2}$ (8CT3) aligned by gate domain. AE2$_{IF/PIP2}$ and AE1$_{OF/PIP2}$ are colored in green and grey, respectively.

b Structural alignment of the core domains of AE2$_{IF/PIP2}$ and AE1$_{OF/PIP2}$. AE2$_{IF/PIP2}$ and AE1$_{OF/PIP2}$ are colored in green and grey, respectively.

c Structural comparison of the TMD of AE2$_{IF/PIP2}$ and AE2$_{inter}^{basic\text{-}KNO3}$ (PDB ID: 8GVA) aligned by the gate domain. Core and gate domain of AE2$_{IF/PIP2}$, AE2$_{inter}^{basic\text{-}KNO3}$ and PIP$_2$ are colored in green, blue, grey and yellow, respectively.

d-e Structural comparison of the TMD (**d**) and the substrate coordination site (**e**) of AE2$_{IF/PIP2}$ and AE2$^{acidic\text{-}KNO3}$ (PDB ID: 8GVH) aligned by the gate domain. The core and gate domain of AE2$_{IF/PIP2}$, AE2$^{acidic\text{-}KNO3}$, CHS (8GVH) and PIP$_2$ (AE2$_{IF/PIP2}$) are colored in green, blue, grey, pink and yellow, respectively. Arrows indicated the displacements of the core domains from AE2$^{acidic\text{-}KNO3}$ state to AE2$_{IF/PIP2}$ state.

f-g Representative FSEC traces of wild-type AE2 (**f**) and AE1 (**g**) after heating at 55 °C for 15 min at different pH.

h Thermal stabilization of wild-type AE1 and AE2 at different pH. Normalized mean fluorescence is shown after heating at 55 °C for 15 min. Residual fluorescences of AE1 and AE2 at pH 7.4 after heating are 80.89 % and 54.80 % respectively. The data are presented as mean values ± SD. The number of independent experiments for both AE1 and AE2 is 3.

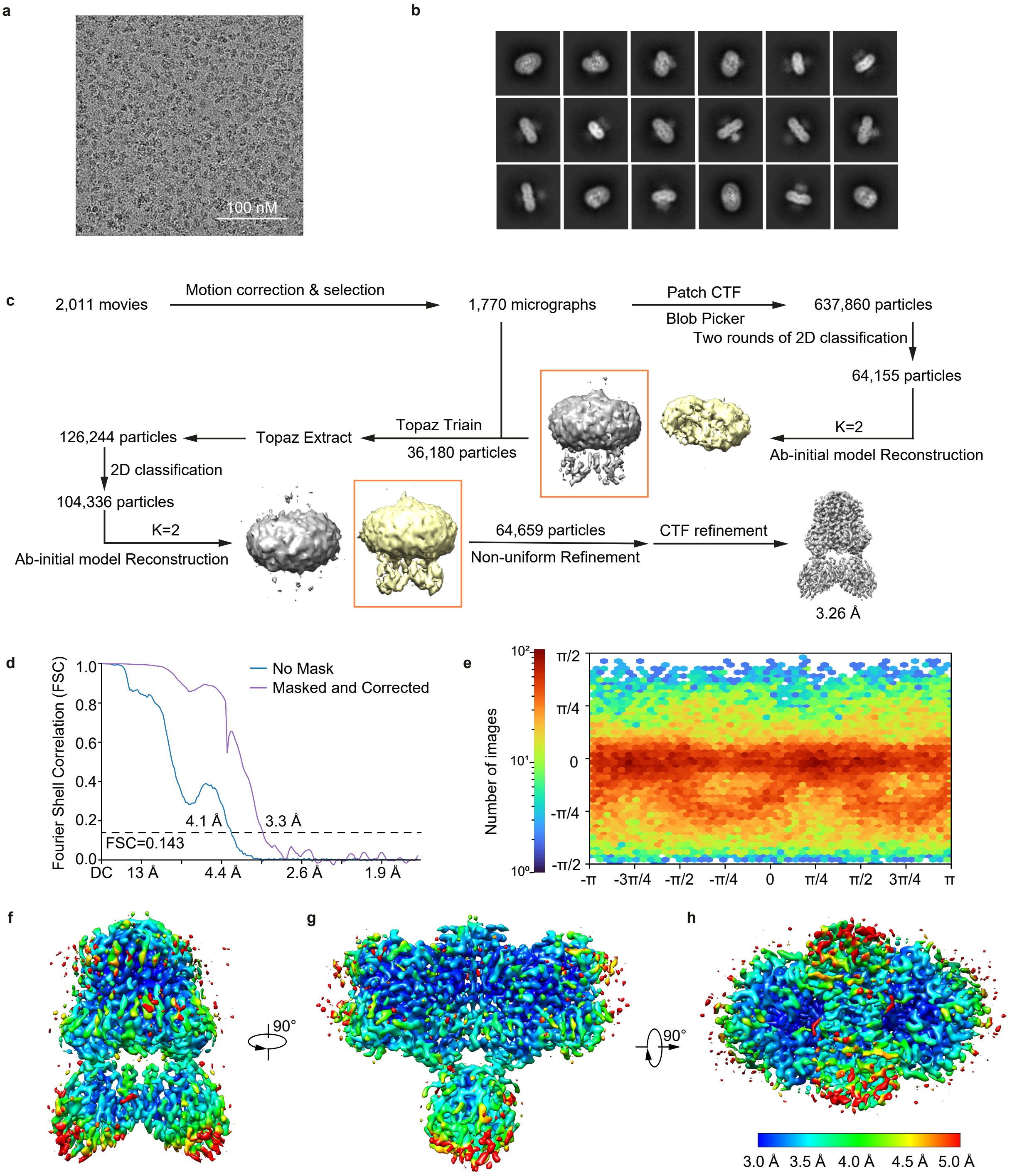

Supplementary Figure. 5: Workflow for cryo-EM data processing of AE2$_{IF/REST}$.

a Representative image from a dataset consisting of 1,770 motion-corrected micrographs.

b Two-dimensional class averages of the sample.

c EM data processing workflow. Two classed generated by ab-initial reconstruction are colored in yellow and grey, respectively. Particles in the class indicated by the orange box were used for finial reconstruction.

d Resolution estimation of the AE2 map, based on the criterion of the FSC 0.143 cut-off.

e Angular distribution of the final reconstruction.

f-h Local resolution maps in different views coloured according to the bar in the lower right corner of the figure.

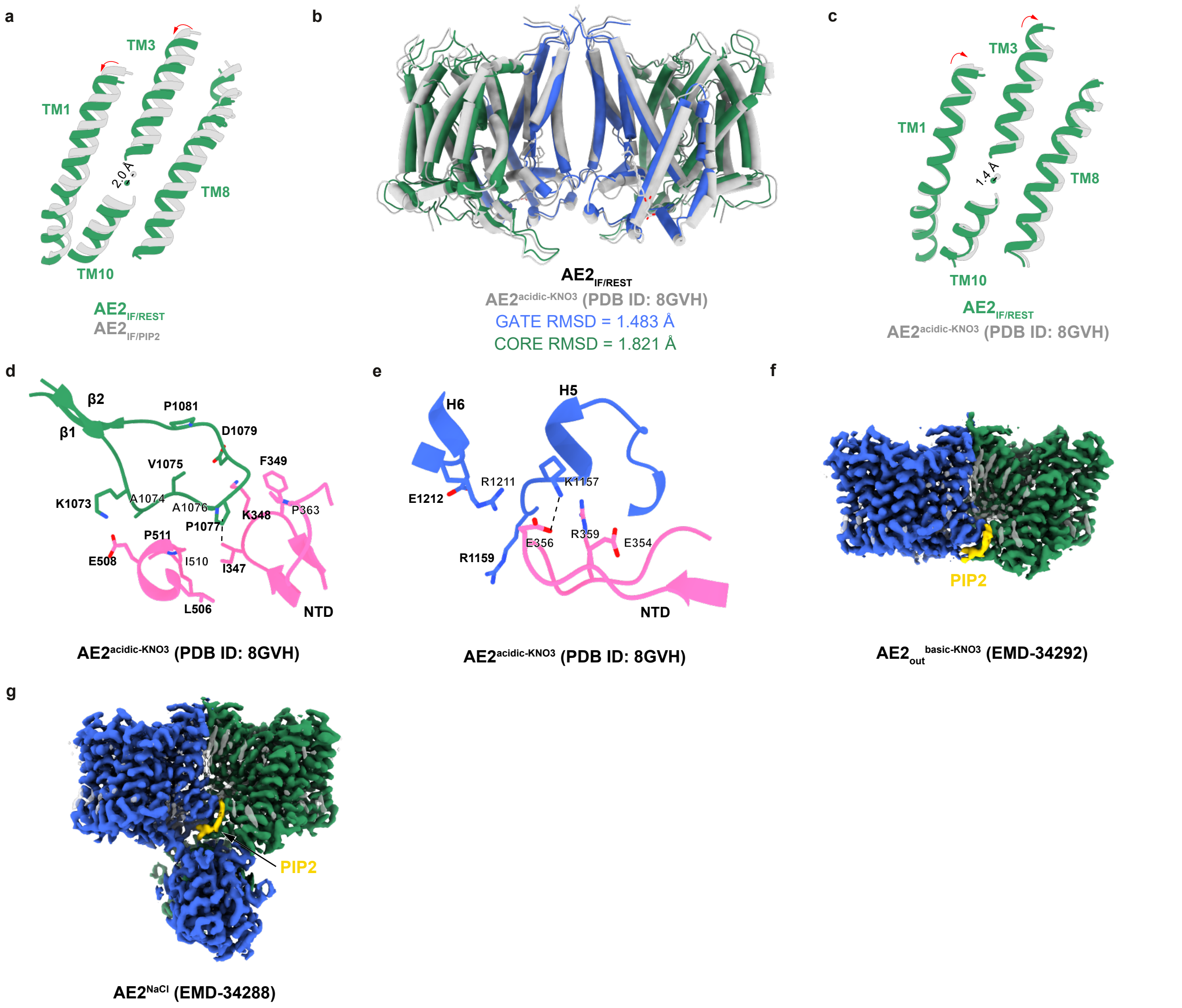

Supplementary Figure. 6: Comparison of the AE2$_{IF/REST}$ structure with AE2 in alternate states.

a Structural comparison of the substrate coordination site of AE2$_{IF/PIP2}$ and AE2$_{IF/REST}$ state aligned by the gate domain. AE2$_{IF/PIP2}$ and AE2$_{IF/REST}$ are colored in grey and green, respectively.

b-c Structural comparison of the TMD (**b**) and the substrate coordination site (**c**) of AE2$_{IF/REST}$ and AE2$^{acidic\text{-}KNO3}$ (PDB ID: 8GVH) aligned by the gate domain. The core and gate domains of AE2$_{IF/REST}$ and AE2$^{acidic\text{-}KNO3}$ are colored in green, blue and grey, respectively. Arrows indicate the displacements of the core domains of the AE2$^{acidic\text{-}KNO3}$ state to AE2$_{IF/REST}$ state.

d-e Interfaces of the AE2$^{acidic\text{-}KNO3}$ NTD and TMD corresponding to Fig. 5f (**d**) and 5g (**e**), respectively. Residues within the interfaces are shown as sticks and interactions between the residues are indicated by dashed lines.

f Map of AE2$_{out}^{basic\text{-}KNO3}$ (EMD-34292). The two protomers, PIP_2 and lipid-like densities are colored in blue, green, yellow and grey, respectively.

g Map of AE2^{NaCl} (EMD-34288). The two protomers, PIP_2 and lipid-like densities are colored in blue, green, yellow and grey, respectively.

Supplementary Table 1

Cryo-EM data collection, refinement and validation statistics

	$AE2_{IF/PIP2}$	$AE2_{IF/REST}$
PDB ID	8JNI	8JNJ
EMDB ID	36448	36449
**Data collection and processing**		
Magnification	165,000 ×	165,000 ×
Voltage (kV)	300	300
Electron exposure (e^{-}/Å^2)	50	50
Defocus range (μm)	-1.5 to -2.0	-1.5 to -2.0
Pixel size (Å)	0.821	0.821
Symmetry imposed	*C2*	*C2*
Initial particle images (no.)	505,650	637,860
Final particle images (no.)	107,451	64,659
Map resolution (Å)	3.2	3.3
FSC threshold	0.143	0.143
Map resolution range (Å)	250-3.2	250-3.3
**Refinement**		
Initial model used (PDB code)	8CT3	8CT3
Model resolution (Å)	3.1	3.2
FSC threshold	0.143	0.143
Model resolution range (Å)	250-3.1	250-3.2
Map sharpening *B* factor (Å^2)	-94.7	-84.1
Model composition		
Non-hydrogen atoms	7,842	11,736
Protein residues	994	1,516
Ligands	4	0
B factors (Å^2)		
Protein	35.13	54.19
Ligand	61.96	
R.m.s. deviations		
Bond lengths (Å)	0.005	0.005
Bond angles (°)	0.982	0.955
Validation		
MolProbity score	1.83	1.52
Clashscore	6.66	7.26
Poor rotamers (%)	1.93	1.45
Ramachandran plot		
Favored (%)	96.33	98.40
Allowed (%)	3.67	1.60
Disallowed (%)	0.00	0.00
Model content	673-855	320-431
	894-1071	503-638
	1086-1221	673-854
		894-1221
