# Supplementary material for: Structural and functional insights into the lipid regulation of human anion exchanger 2
Source: Nat Commun. 2024 Jan 26;15:759. doi: 10.1038/s41467-024-44966-0 (PMC10810954; doi:10.1038/s41467-024-44966-0)
Supplement: Supplementary file 3 — Reporting Summary [file 41467_2024_44966_MOESM3_ESM.pdf]

nature portfolio

Corresponding author(s): Yuxin Yin

Last updated by author(s): Dec 18, 2023



# Reporting Summary

Nature Portfolio wishes to improve the reproducibility of the work that we publish. This form provides structure for consistency and transparency in reporting. For further information on Nature Portfolio policies, see our Editorial Policies and the Editorial Policy Checklist.

## Statistics

For all statistical analyses, confirm that the following items are present in the figure legend, table legend, main text, or Methods section.

| n/a | Confirmed | |
|---|---|---|
| ☐ | ☒ | The exact sample size (*n*) for each experimental group/condition, given as a discrete number and unit of measurement |
| ☐ | ☒ | A statement on whether measurements were taken from distinct samples or whether the same sample was measured repeatedly |
| ☐ | ☒ | The statistical test(s) used AND whether they are one- or two-sided<br>*Only common tests should be described solely by name; describe more complex techniques in the Methods section.* |
| ☒ | ☐ | A description of all covariates tested |
| ☒ | ☐ | A description of any assumptions or corrections, such as tests of normality and adjustment for multiple comparisons |
| ☐ | ☒ | A full description of the statistical parameters including central tendency (e.g. means) or other basic estimates (e.g. regression coefficient) AND variation (e.g. standard deviation) or associated estimates of uncertainty (e.g. confidence intervals) |
| ☐ | ☒ | For null hypothesis testing, the test statistic (e.g. *F*, *t*, *r*) with confidence intervals, effect sizes, degrees of freedom and *P* value noted<br>*Give P values as exact values whenever suitable.* |
| ☒ | ☐ | For Bayesian analysis, information on the choice of priors and Markov chain Monte Carlo settings |
| ☒ | ☐ | For hierarchical and complex designs, identification of the appropriate level for tests and full reporting of outcomes |
| ☒ | ☐ | Estimates of effect sizes (e.g. Cohen's *d*, Pearson's *r*), indicating how they were calculated |

*Our web collection on statistics for biologists contains articles on many of the points above.*

## Software and code

Policy information about availability of computer code

| | |
|---|---|
| Data collection | Cryo-electron microscopy: Serial-EM-3.6.11; images for anion exchange analysis: Zen 2.3 (black edition, Carl Zeiss); flow cytometry: FACSDiva™ Software v6.1 (BD Bioscience); |
| Data analysis | cryoSPARC software-3.1.0, Topaz-0.2.5, Alphafold, PHENIX-1.19.2-4158, Coot-0.9.2, UCSF Chimera-1.14, Pymol-1.7.0.5, UCSF ChimeraX-1.5, Flow Jo v10.6.2, Carl Zeiss Zen 2.3 (blue edition), Microsoft® Excel® 2021MSO, IBM SPSS Statistics v27.0.0.0, GraphPad Prism v9.3.0. |

For manuscripts utilizing custom algorithms or software that are central to the research but not yet described in published literature, software must be made available to editors and reviewers. We strongly encourage code deposition in a community repository (e.g. GitHub). See the Nature Portfolio guidelines for submitting code & software for further information.

## Data

Policy information about availability of data

All manuscripts must include a data availability statement. This statement should provide the following information, where applicable:

- Accession codes, unique identifiers, or web links for publicly available datasets
- A description of any restrictions on data availability
- For clinical datasets or third party data, please ensure that the statement adheres to our policy

Cryo-EM maps and atomic coordinates are deposited in the Electron Microscopy Data Bank (EMDB) and RCSB Protein Data Bank (PDB) as follows: AE2IF/PIP2 : EMD-36448 [https://www.ebi.ac.uk/pdbe/entry/emdb/EMD-36448] and PDB: 8JNI [https://doi.org/10.2210/pdb8JNI/pdb]; AE2IF/REST: EMD-36449 [https://

www.ebi.ac.uk/pdbe/entry/emdb/EMD-36449] and PDB: 8JNJ [https://doi.org/10.2210/pdb8JNJ/pdb]. 8CT3 [https://doi.org/10.2210/pdb8CT3/pdb], 8GVA [https://doi.org/10.2210/pdb8GVA/pdb]. 8GVH [https://doi.org/10.2210/pdb8GVH/pdb], EMD-26143 [https://www.ebi.ac.uk/pdbe/entry/emdb/EMD-26143], EMD-34292 [https://www.ebi.ac.uk/pdbe/entry/emdb/EMD-34292] and EMD-34288 [https://www.ebi.ac.uk/pdbe/entry/emdb/EMD-34288] are already available on Protein Data Bank.

## Research involving human participants, their data, or biological material

Policy information about studies with human participants or human data. See also policy information about sex, gender (identity/presentation), and sexual orientation and race, ethnicity and racism.

| | |
|---|---|
| Reporting on sex and gender | N/A |
| Reporting on race, ethnicity, or other socially relevant groupings | N/A |
| Population characteristics | N/A |
| Recruitment | N/A |
| Ethics oversight | N/A |

Note that full information on the approval of the study protocol must also be provided in the manuscript.

# Field-specific reporting

Please select the one below that is the best fit for your research. If you are not sure, read the appropriate sections before making your selection.

☒ Life sciences ☐ Behavioural & social sciences ☐ Ecological, evolutionary & environmental sciences

For a reference copy of the document with all sections, see nature.com/documents/nr-reporting-summary-flat.pdf

# Life sciences study design

All studies must disclose on these points even when the disclosure is negative.

| | |
|---|---|
| Sample size | All of functional experiments were repeated at least 3 times and sample sizes were indicated in figure legends. The sample sizes were chosen based on the standard and common practices in the field, ensuring the reproducibility of the experiments. No statistical methods were used to predetermine the sample size. The sample size was adequate based on the distribution of data and clearly visible effects. |
| Data exclusions | Cryo-EM micrographs with ice or ethane contamination, empty carbon, and poor CTF fit (> 5 Å) were excluded manually. Particles belonging to bad classes were discarded and the data processing flowchart were summarized in Supplementary Figures. These criteria were pre-established and the procedure is a common practise in cryo-EM image analysis. |
| Replication | All attempts at replication were successful according to the detailed protocols described in the methods section. The numbers of replication were described in figure legends. |
| Randomization | For cryo-EM 3D refinement, all particles were randomly split into two groups. No group allocation was needed for functional experiments in this study. |
| Blinding | The investigators were blinded to group allocation during cryo-EM half map generation. Blinding is not relevant for protein structure determination and functional assays because these results are not subjective. |

# Reporting for specific materials, systems and methods

We require information from authors about some types of materials, experimental systems and methods used in many studies. Here, indicate whether each material, system or method listed is relevant to your study. If you are not sure if a list item applies to your research, read the appropriate section before selecting a response.

## Materials & experimental systems

| n/a | Involved in the study |
|---|---|
| ☐ | ☒ Antibodies |
| ☐ | ☒ Eukaryotic cell lines |
| ☒ | ☐ Palaeontology and archaeology |
| ☒ | ☐ Animals and other organisms |
| ☒ | ☐ Clinical data |
| ☒ | ☐ Dual use research of concern |
| ☒ | ☐ Plants |

## Methods

| n/a | Involved in the study |
|---|---|
| ☒ | ☐ ChIP-seq |
| ☐ | ☒ Flow cytometry |
| ☒ | ☐ MRI-based neuroimaging |

# Antibodies

| | |
|---|---|
| Antibodies used | PE anti-HA.11 Epitope Tag Antibody (1:500 dilution, BioLegend, Cat # 901518, clone 16B12) |
| Validation | PE anti-HA.11 clone 16B12: FC, ICFC; https://www.biolegend.com/en-us/punchout/punchout-products/product-detail/pe-anti-ha-11-epitope-tag-antibody-13535 |

# Eukaryotic cell lines

Policy information about cell lines and Sex and Gender in Research

| | |
|---|---|
| Cell line source(s) | Sf9 and HEK293F cells were purchased from Thermo Fisher Scientific. HEK293T cell was purchased from ATCC. |
| Authentication | None of the cell line used was authenticated. |
| Mycoplasma contamination | All cell lines were tested negative for mycoplasma contamination. |
| Commonly misidentified lines (See ICLAC register) | No commonly misidentified cell lines were used. |

# Flow Cytometry

## Plots

Confirm that:

☒ The axis labels state the marker and fluorochrome used (e.g. CD4-FITC).

☒ The axis scales are clearly visible. Include numbers along axes only for bottom left plot of group (a 'group' is an analysis of identical markers).

☐ All plots are contour plots with outliers or pseudocolor plots.

☒ A numerical value for number of cells or percentage (with statistics) is provided.

## Methodology

| | |
|---|---|
| Sample preparation | 5x10^5 HEK293 cells were seeded in a well of 12-well plate and were grown overnight at 37 °C and 5% CO2 in a humidified incubator. Cells in each well were transfected with 1 µg of PBM plasmids encoding the HA tag-inserted C-terminally GFP-tagged AE2 or mutants using PEI. 24 h post-transfection, cells were washed twice with ice cold PBS, resuspended and pipetted into single-cell suspension, and pelleted via centrifugation at 300 xg for 2 min at 4 °C for the following antibody incubation. For surface staining, cell pellet from each well was resuspended in 50 µl PBS supplemented with 1% FBS and PE anti-HA.11(1:500, BioLegend) at 37 °C for 30 min. Subsequently, cells were washed twice with ice cold PBS and were eventually resuspended in 500 µl ice cold PBS. |
| Instrument | Cells were analyzed using a BD LSRFortessa cell analyzer with a 488nm laser and 585/40 bandpass filter. |
| Software | Data were collected using BD FACSDiva™ Software. Data were analyzed using Flow Jo v10.6.2. |
| Cell population abundance | 10^5 singlets were collected for each sample. |
| Gating strategy | Live single cells were identified by cell size and granularity in a FCS/SSC plot. Gates indicating boundaries between positive and negative were based on the control sample staining. Expression of target proteins were evaluated on these populations as indicated in the figures and methods. |

☒ Tick this box to confirm that a figure exemplifying the gating strategy is provided in the Supplementary Information.
