# Supplementary material for: Structural and functional insights into the lipid regulation of human anion exchanger 2
Source: Nat Commun. 2024 Jan 26;15:759. doi: 10.1038/s41467-024-44966-0 (PMC10810954; doi:10.1038/s41467-024-44966-0)
Supplement: Supplementary file 2 — Peer Review File [file 41467_2024_44966_MOESM2_ESM.pdf]

nature portfolio

Peer Review File

Structural and functional insights into the lipid regulation of human anion exchanger 2

Open Access This file is licensed under a Creative Commons Attribution 4.0 International License, which permits use, sharing, adaptation, distribution and reproduction in any medium or format, as long as you give appropriate credit to the original author(s) and the source, provide a link to the Creative Commons license, and indicate if changes were made. In the cases where the authors are anonymous, such as is the case for the reports of anonymous peer reviewers, author attribution should be to 'Anonymous Referee' followed by a clear attribution to the source work. The images or other third party material in this file are included in the article's Creative Commons license, unless indicated otherwise in a credit line to the material. If material is not included in the article's Creative Commons license and your intended use is not permitted by statutory regulation or exceeds the permitted use, you will need to obtain permission directly from the copyright holder. To view a copy of this license, visit http://creativecommons.org/licenses/by/4.0/.

REVIEWER COMMENTS

Reviewer #1 (Remarks to the Author):

This work presents a 3.2A cryo-EM structure of the hAE2 TMD in complex with PIP2, and a 3.3A holo-AE2 structure in the absence of PIP2. Binding of PIP2 is claimed to be essential for AE2-mediated anion exchange activity. Triple missense mutation of residues surrounding the PIP2 binding site is shown to alter TM7 conformation and stabilize interaction between TMD and NTD.

The work appears to be of high quality, but has problems in written presentation described below.

The prior contributions of ref. 40 and its differences with the present work need to be acknowledged and summarized in the Introduction, rather than waiting until Discussion as is currently the case. In Discussion, the authors need to compare and if possible to integrate their new finding of PIP2 binding with the Ref. 40 description of regulation by the AE2 Cterminal tail.

Abstract:

Line 33: “the pH sensor function of PIP2 molecules” should be changed to “the central contribution of PIP2 to the function of AE2”. This paper does not explore regulation of AE2 activity by either intracellular or extracellular pH. The relationship between PIP2 binding and the the PIP2 binding pocket side chains with pH is not adequately developed.

Intro:

General: The introduction focuses on the regulation of AE2 by pH. However, the experiments presented in this paper do not really address regulation by pH.

Lines 59-70. The previously published structure of hAE2 (ref 40) should be cited here, along with the other SLC4 structures noted in lines 61-64. Moreover, the central findings of ref 40 ought to be cited here, as they appeared in print several months before submission of this manuscript.

Major points.

AE2 structure:

Text does not mention whether the structure shown is in inward-facing or outward-facing conformation, and whether the two protomers are in the same conformation. These needed comments should also compare the conformations presented in the current work to those presented in ref. 40, emphasizing what’s new here, what is confirmatory, and what is different.

P 4 line 94 defines Fig 1a-c structure as AE2IF/PIP2. Is "IF" intended to mean "inward-facing?" Definition is needed here – meaning becomes clear only later in text.

Fig. S2d appears to show an inter-protomeric disulfide linkage between neighboring C855 residues. This would be a novel finding for AE2 (although not for SLC4A4), but neither text nor legend discusses it. This needs mention and explanation in main text. Discussion should note this disulfide linkage as a point of regulation either during biosynthetic trafficking or function at the cell surface. (If I misinterpret the structure in Fig S2d, I would suggest presentation of a different view of the structure so as to Perhaps the orientation of the structure can be changed if I have misinterpreted this figure.)

PIP2 binding site:

All figures with AE1 annotated as (PDB ID: 8CT3) should be accompanied at first mention in each legend by reminders in the corresponding legends that this structure is outward-facing.

Fig 2a is cited as showing TM7 and HL forming the PIP2 binding site, but this is not interpretable from the figure – it should be annotated with TM7 and HL, or an additional panel (perhaps ribbon or stick) should be added to illustrate the point.

Line 97 states that the AE2 PIP2 binding site is consistent with the AE1 PIP2 binding site, but doesn't mention that AE1 binds adjacent PIP2 and cholesterol molecules, each on one protomer at the interprotomeric cytoplasmic interface. Moreover, Zhang et al (ref 40) find incorporation of cholesteryl hemisuccinate bound in a position close to the cytosolic interprotomeric surface.

Cholesteryl hemisuccinate was present during solubilization in the current study, but appears to be absent from the current AE2 structure.

Can cholesterol be docked into the current AE2 structure or any of the previous structures?

Does the absence or presence of PIP2 or of cholesterol reflect the conditions of solubilization for both AE2 and AE1 in the current work, ref 20 and ref 40?

Line 104: This last sentence of p. 4 should be followed by mention of previous AE1 structures in which bound PIP2 was not detected (or not recognized).

(Methods: lines 255-6: supplemented with how much additional LMNG and CHS?)

Mutagenesis of PIP2-interacting residues:

In Fig 3B, the student t -test seems inappropriate – the data should be evaluated by ANOVA with pair-wise post-hoc comparisons to WT. The normalized individual flux values for WT should also be shown. Legend or text should specify absolute value of mean +/-sd WT AE2-mediated Cl--driven base flux. Fig 3Sk and Fig 3Sl should also be evaluated by ANOVA, and not by t-test. Fig. 3Sk is redundant to Fig 3Sl, which provides more meaningful information, rather than a binary determination of surface AE2 positivity or negativity such as shown in Fig. 3Sk.

All experiments of the mutants presented in Fig 3g and Fig 3h should be added as bars to Fig 3b. If numbers don't suffice, n should be increased.

Authors report but don't discuss the (perhaps unexpected) finding that H1148Y mutation (to the sequence of PIP2-binding AE1) reduces AE2 activity as much as do nonconservative mutations to other PIP2 binding residues.

PIP2 binding is described as "essential to AE2 activity." Indeed, the triple mutant (R932A/H1147A/H1148A) is expressed at the cell surface as well as multiple single PIP2 site mutants, but unlike the 50% reduction in anion exchange activity of the latter, loses all transport activity. And citation of previous mAE2 H1144 mutation may also be consistent with the importance of PIP2 binding. However, at this stage of the text, "essential" is overstatement. The mutatgenesis of these residues later in the text constitutes the required experiment to comment further on the possible function of PIP2 binding.

Line 127: The H1144 mutation result cited "suggests," but does not "indicate," that PIP2 participates in regulation of AE2 activity by pH.

Pp 5-6 concerns comparison of PIP2-bound AE1 structure from ref 20 with the current AE2IF/PIP2 structure. However, inward-facing structures have been published by Zhang et al (ref 40, not mentioned until late in Discussion). These outward- and inward-facing structures must be compared as well, either here or in the next section "Interactions between NTD and of AE2 in absence of PIP2." If current Fig 4a-d is kept, then comparison of current PIP2-bound structure with Zhang et al AE2 structure lacking PIP2 should be added as a supplemental figure, or at least the Gate domain RSMD with that of the published inward-facing Gate domain lacking PIP2 but containing nearby cholesteryl hemisuccinate should be reported. In particular, the interprotomeric subunit interface of ref 40 should be compared to that found in the current work.

Fig. 4b-c legend should specify "one protomer of AE1 TMD (grey) and AE2 TME (colored)……The shift direction of the transmembrane helices from the outward-facing state (of AE1-TMD) to the inward-facing state (of AE2 TMD)….

Fig S5b requires addition of TM annotations.

Fig 4e should add yellow color to uncharged portions of PIP2. DDG's for PIP2 binding should be compared.

Fig. 4g text treats data as two indistinguishable melting curves. However, the curves (with remarkably tight variance) appear to this reviewer's eye to differ significantly with a [illegible]Tm of ~5oC. Therefore Fig. 4g or related text should include F-test or other statistical comparison of the two melting curves. Such a conclusion would suggest some contribution of conformational change by substitution of charged and polar residues with neutral and smaller Ala.

Fig. 5 legend needs to define AE2IF/REST, since reader encounters the legend prior to text definition on p 7 lines 162-4.

p. 7 line 165 "inward-facing rotation" should probably be better described as "inward rotational displacement"?

Fig 5d legend is incorrect in describing red arrows as indicating the transition from outward-facing to inward-facing state. They rather seem to indicate the computational difference between the IF/PIP2 and IF/REST states of AE2, as noted in text (p.7 lines 164-6).

p. 7 lines 166-7: Absence of PIP2 from the AE2IF/REST state would be better displayed if the two PIP2 molecules in panel were differentiable by color – perhaps one could have a yellow backbone and the other an orange backbone, with appropriately annotated legends for panels c and d?

Fig. 5e should add annotation of the mutated residues 932, 1147 and 1148 in both "AE2IF/REST and AE2IF/PIP2 chains.

Fig 5f legend should specify that the partial structure shown is from (and visible only in) AE2IF/REST.

Fig 5f should be modified to indicate the specific charge-pairs, and at least some of the proposed hydrophobic interactions between TMD loop 10-11 and stabilized NTD, as described in lines 172-5. Same should be done in Fig. 5g. These interactions should be directly compared to the TMD-NTD interface revealed in Fig 2 of ref. 40.

Mutation of the NTD region shown at right in Fig 5f acid shifts the pH vs. activity curve (increases activity over a range of pH values) whereas further deletion of the downstream NTD region shown in Fig 5g can rescue normal pH-sensitive activity. How does these earlier findings relate to the current results?

Lines 185-6. "…even on the SLC4 family transporters" sounds inappropriately hypothetical, as PIP2 has already been published as an activity regulator of SLC4A4 and SLC4A7 variants. Please update and revise. Ref 36 recognizing this for SLC4A4 is currently not mentioned until Discussion.

Fig 6c should remove the yellow PIP2 circle entirely for clarity -or perhaps change its color to grey.

Discussion:

Lines 195-8 claim that PIP2 disrupts the interaction between TMD and NTD, preventing inhibition by NTD of AE2 conformational transition and promoting substrate transport. This is overstated. The study does show that a triple mutant that disrupts PIP2 binding stabilizes the TMD-NTD interaction and is inactive as a transporter. That experiment indeed suggests, but does not prove, that PIP2 binding is required for anion transport. However, direct functional evidence of NTD inhibitor interaction with TMD is not demonstrated here. Authors should test the effects of PIP2 depletion vs supplementation on activity of wildtype AE2 in intact cells or in proteoliposomes of defined lipid compositions.

Authors should summarize previous work on regulation of red cell AE1 activity by cholesterol depletion and supplementation. The authors should also link the current work to previous biophysical studies comparing the effects of cholesterol and PIP2 on AE1 (+/- glycophorin A) in lipid bilayers.

Not addressed at all in the paper is the difference between the currently proposed regulation of activity by PIP2, and the previously proposed regulation of activity by insertion of the AE2 C-terminal tail into the inner vestibule detected in the low pH inward-facing structure (ref 40).

Can PIP2 be detected or be docked into any of the reported AE2 structures of ref. 40?

The Fig 6 model is unclear about the meaning of the green tail of the Core domain. Is this tail the TMD C-terminus? Or is it the section of the TM10-11 cytoplasmic loop that is stabilized by PIP2? If the former, then the role of the C-terminus is incorporated into the Fig. 6 model without any evidence in the current paper and without clear attribution to ref 40.

How can PIP2 be essential for AE2 function when AE2 functions in the apparent absence of PIP2 in ref 40? Was PIP2 present but undetected in some of the structures of ref 40? Were detergent solubilization conditions and imaging conditions sufficiently different to account for the absence of presence of PIP2? The Fig 6a schematic with PIP2 at the interprotomeric interface shows each protomer's inner vestibule apparently blocked by its own C-terminal peptide – was such a structure present among the image class-averages of cryo-EM particles, but not reported here?

Do PIP2 and the C-terminal peptide compete for the same binding site in the inner vestibule? Do they exert the same or different effects on transport activity?

Does inter-protomeric PIP2 characterize only the inward state of AE2? Could PIP2 be unrelated or secondarily related to the outward-inward transition, rather controlling anion unbinding from the inward-facing AE2 under conditions permitting release to the cytoplasm?

Methods:

Line 265: Define buffer and salt composition of concentrated AE2 sample deposited on grid for cryo-EM.

Line 277: Define CTF as Contrast Transfer Function

Line 285 "as previously described" – cite ref or restate as "as described above"

Line 295: clarify that cells for flow cytometry were transfected with HA-tagged AE2 carrying C-terminal GFP, if that was the case.

Statistics: Line 325 mentions standard error, but all legends mention standard deviation. Since v

Minor points

English syntax and grammar need correction throughout manuscript

Throughout manuscript, abbreviations are introduced without definition. This should be remedied.

Fig S1 legend: a) "motion-corrected"; c) provide some detail describing blue, gray and yellow low-res structures and meaning of the orange box; f-h: provide descriptions of the three views shown, and explanation of the lower red (low res) structures at the bottom of panels f and g

Fig 1 legend: a and b) define gray lipids. d) define residue # at N-terminus of N[illegible]1. Define residue #s at C-terminus of resolved region beyond NH8 and N-terminus of resolved region preceding H1.

Fig. S2d legend or relevant text should list the interacting inter-protomeric residue pairs. Fig S2d shows an inter-protomeric disulfide linkage between C855 of each protomer, but this is not mentioned in the relevant text on p 4 lines 89-90. Fig S2e legend should specify that image of putative chloride binding site is from an inward-facing form, since the coordination site was accessible from the cytoplasmic side, as described in text.

Fig 2b should show the AE2 interacting residue mesh in blue rather than gray, which is hard to see adjacent to the yellow mesh, even enlarged 4-fold.

Figs S3k and S3l need X Axis labels either in place of or to supplement the current color keys, which are difficult to read. The colored circles must be larger, in addition to labeling the columns along the X axis using angled labels.

Reviewer #2 (Remarks to the Author):

This is an impressive cryo-EM study that reports on the 3.2 A structure of PIP2 binding to the inward-facing configuration of partial AE2 (TMD). The authors extend their analysis and description of PIP2 binding by also solving the structure of AE2 unbound to PIP2, and the 2 structures provide insight into the role of the N terminal domain (NTD) and associated structural changes. Further support for the proposed structures and models is provided my mutagenesis data of the PIP2-binding site highlighting its impact on function, and comparative analysis to recent work by Vallese et al. identifying PIP2 binding to AE1. This in an important study that provides insight into a regulatory mechanism of a membrane acid-base transporter, and the findings will help the field understand such regulation of other related bicarbonate transporters, and how it may link to their pH sensing mechanism(s). A number of issues need to be addressed.

1. The current emphasis on PIP2 binding/regulation being linked to the protein's pH sensor is too strong with the limited data. pH sensing is not examined in the current study. This aspect needs to be toned down in the abstract and elsewhere.

2. There is discussion on p. 4 about the "flexibility" and possible "unstable" nature of NTD in the structural analysis. How can the authors then be confident in their assessment of the PIP2-sensitive positioning of the NTD in their model proposed in Fig. 6.

3. Fig. 3 functional analysis. Based on the associated localization data (Suppl. Fig. 3), these mutations have effects on both localization and/or function. When factoring in a decrease in expression for R932 and H1148 mutations, for example, there appears to be a decrease in expression by ~25%, so the subsequent functional decrease of ~25% appears quite mild. This needs to be made clear and discussed.

Also, the Fig. 3b analysis doesn't take into account the pHi sensitivity of the measurements. For example, the re-addition of Cl- in experiments g and h may have had no acidifying effect because the pHi was already low. This needs to be discussed. In theory, the pHi values could be low because of reduced activity of the AE2 to begin with. However, based to the previous paragraph, would a 50% decrease in activity/expression account for this? It might be worth looking at the resting pHi of each of these mutants to help in proposing some explanations.

In light of these points above, it seems premature to conclude that any of these mutations "disrupted the substrate coordination site"

4. It is not clear why the authors conclude that the difference in traces in Fig. 4g doesn't represent a thermal shift.

5. Overall, this is a well-written and logically presented story. However, grammar needs to be improved in many places.

Reviewer #3 (Remarks to the Author):

In the manuscript "Structural insights into the lipid regulation of human anion exchanger 2" Zhang et al determine cryoEM structures of AE2 and identify a PIP2 binding site that is important for transport function. Through structural and functional analyses, Zhang et al show that disruption of the PIP2 binding site causes conformational rearrangements within the TMD that promote association with the NTD. Based on their work the authors propose that PIP2 allosterically activates AE2 for substrate transport. The presented work complements previous structural studies on AE2, and provides much needed structural insight into the modulation of transporters by membrane components such as PIP2. The presented work is interesting, but a confusing discussion/summary cartoon, lack of comparison with previous work, and incomplete modeling/interpretation of the PIP2 site dampens enthusiasm. Several major points need to be addressed:

• The cartoon shown in Figure 6 is confusing and not very helpful to understand the role of PIP2 in modulation of transporter activity: The NTD is outlined with dashed lines in all panels. The difference between bound and unbound PIP2 is only indicated by solid and dashed lines rather than removing the molecule entirely. The legend states "In the presence of PIP2, substrates binding sites is accessible to Cl- in the outward-facing conformation and to HCO3- in the inward facing conformation", but the authors provide a Chloride- and PIP2-bound IF state structure. So none of the states proposed here are backed

up by structures in this manuscript. The authors also discuss a pH-sensing role of PIP2, which is missing from this cartoon. Overall, this figure is important to understanding the findings reported herein and needs to be much improved.

• The density for the modeled PIP2 atoms is excellent, but the PIP2 molecule is not complete – much of the acyl chain is missing. The authors need to state that in the manuscript, as it is currently implied that the whole molecule was built. Also, the cryoEM density shows that there are other membrane components involved in modulating AE2 (and perhaps PIP2) activity, but PIP2 is the only modeled lipid. Given the focus on lipid modulation in this work, some of these should at least be modeled, particularly as previous work identifies many lipids and sterols in the modulation of SLC4 transporters and AE2. There are two additional lipid-like molecules in the immediate vicinity of PIP2 that are likely the most relevant to the subject of the paper. One of them directly packs against the acyl-chain of PIP2 and likely directly impacts PIP2 affinity and function. Previous work of ion channels (e.g. PMID: 34433062 for ion channels) has structurally elucidated how sterols contribute to PIP2 binding. Something similar needs to be done here to define the PIP2 pocket in detail.

• Related to the previous point, the PIP2 density figure (Supplementary Figure 2f) needs to show density for the nearby residues of the binding pocket to provide an assessment of model quality of the sidechains involved. Within this context, one should also show the neighboring lipid/sterol density that plays a role in coordinating PIP2.

• Previous work reported six AE2 structures, and this work should already be cited in the introduction in line 62, and not just later. Despite these structures, the authors do not perform any structural comparison with the previous AE2 states, which could have provided novel insights and validated the author's work. For instance, overlay with 8GV9 shows a very different positioning of the Chloride ion compared to the herein presented TMD structure. Since the herein reported Chloride ion is more than 4.5 A from R1060 and does not agree with previously reported work (Zhekova et al, Capper et al), this could also be water, other buffer components, or noise from the map. At the very least, the authors would have to explain the discrepancy to previous work. Similarly, the manuscript could benefit from comparison to other structural AE2 states. For instance, what does the PIP2 binding site look like in the OF state? How do basic or acidic conditions change the conformation of the PIP2 binding site? These analyses would greatly strengthen this work.

• There are several peculiar details in the flux data. While overall consistent with the author's hypothesis, only WT AE2 displays a stable pH baseline before addition of chloride. For instance, cellular pH of the R932A mutant drops by 0.4 units before the authors even add Chloride. That raises several questions, did the authors not provide sufficient time to equilibrate the system, or do the mutant cause ion leakage? Why do the recordings of the triple mutant and the R1060A mutant start at pH 6.9 compared to the other experiments that start at cellular pH? I would also recommend to expand the

alignment in Figure 3i to include the complete family of SLC4 transporters as the authors later make a sweeping statement about PIP2 pockets being potentially present in all SLC4 transporters.

• There are numerous typos and grammatical errors throughout the manuscript, and several statements are awkwardly phrased. While there are too many to list all of them, here are some that need clarification:

o Line 46/47: “Alternative promoters of the SLC4a2 gene give rise to three AE2 isoforms…” It sounds like three different promoters can produce 3 different isoforms from the same coding sequence.

o Line 76: “SEC” is not defined

o Line 84/85: “..suggesting that [the] NTD was unstable..” This implies that the NTD unfolds or aggregates. I suppose the authors want to state that the NTD was too flexible to assume a conformation detectable by cryoEM.

o Line 120/121: “Phylogenetic analysis revealed that residues consist of the PIP2 binding site are absolutely conserve among AE2 orthologs…” There is no phylogenetic analysis in this manuscript, only sequence alignment of the PIP2 pocket. “absolutely” needs to be removed, and several typos need to be fixed in this section.

o Line 136/137: “[The] core domain of AE2IF/PIP2 rotates towards the gate and domain and [shifts] downward to the cytosolic side …”

o Line 176/177: “Ankyrin complexes solved by others display extensive interfaces between AE1 and protein 4.2 indicating a strong relationship between these proteins.” “relationship” is awkward in this context. Rephrase!

o Line 188: “Although PIP2 is a minority phospholipid…” “Minority” is awkward in this context, and it doesn’t matter that it isn’t the main constituent of the membrane. Remove!

o Line 202/203: “mutating R932, K1147 and H1148 has (have) little impact on the

structural stability and plasma membrane trafficking.” Supplementary figure 3l shows that the triple mutant reduces membrane expression by ~30%, so this statement needs to be corrected.

Point-by-point response to reviewers

We are grateful for the thorough evaluation of our manuscript from the reviewers. A detailed point-by-point response to all comments and suggestions of the reviewers are provided in this document.

Reviewer #1 (Remarks to the Author):

This work presents a 3.2A cryo-EM structure of the hAE2 TMD in complex with PIP2, and a 3.3A holo-AE2 structure in the absence of PIP2. Binding of PIP2 is claimed to be essential for AE2-mediated anion exchange activity. Triple missense mutation of residues surrounding the PIP2 binding site is shown to alter TM7 conformation and stabilize interaction between TMD and NTD.

The work appears to be of high quality but has problems in written presentation described below.

The prior contributions of ref. 40 and its differences with the present work need to be acknowledged and summarized in the Introduction, rather than waiting until Discussion as is currently the case. In Discussion, the authors need to compare and if possible to integrate their new finding of PIP2 binding with the Ref. 40 description of regulation by the AE2 C-terminal tail.

We thank the reviewer for the thorough evaluation of our work and the thoughtful comments.

Abstract:

Line 33: "the pH sensor function of PIP2 molecules" should be changed to "the central contribution of PIP2 to the function of AE2". This paper does not explore regulation of AE2 activity by either intracellular or extracellular pH. The relationship between PIP2 binding and the PIP2 binding pocket side chains with pH is not adequately developed.

Response: We have revised the text according to reviewer's suggestion in line 33.

Intro:

General: The introduction focuses on the regulation of AE2 by pH. However, the experiments presented in this paper do not really address regulation by pH.

Lines 59-70. The previously published structure of hAE2 (ref 40) should be cited here, along with the other SLC4 structures noted in lines 61-64. Moreover, the central findings of ref 40 ought to be cited here, as they appeared in print several months before submission of this manuscript.

Response: We have reduced the content about the regulation of AE2 by pH in line 59-61, cited the reference of recently determined hAE2 structure in line 62 and described their central findings in line 63-65.

Major points.

AE2 structure:

Text does not mention whether the structure shown is in inward-facing or outward-facing conformation, and whether the two protomers are in the same conformation. These needed comments should also compare the conformations presented in the current work to those presented in ref. 40, emphasizing what's new here, what is confirmatory, and what is different.

Response: Two structures of AE2 we determined are in the inward-facing state with two protomers in the same conformation and we have added these details in line 80-82 and 175-176. Conformations presented in the current work were compared with structures determined by Zhang et al. (Zhang et al., 2023, PMID:37002221) in Supplementary Fig. 4c-e and 6b-e and described in line 153-158 and 187-191 as suggested.

P 4 line 94 defines Fig 1a-c structure as AE2IF/PIP2. Is "IF" intended to mean "inward-facing?" Definition is needed here – meaning becomes clear only later in text.

Response: We have added the definition of $AE2_{IF/PIP2}$ as inward-facing state in complex with PIP_2 in line 99-100 as suggested.

Fig. S2d appears to show an inter-protomeric disulfide linkage between neighboring C855 residues. This would be a novel finding for AE2 (although not for SLC4A4), but neither text nor legend discusses it. This needs mention and explanation in main text. Discussion should note this disulfide linkage as a point of regulation either during biosynthetic trafficking or function at the cell surface. (If I misinterpret the structure in Fig S2d, I would suggest presentation of a different view of the structure so as to Perhaps the orientation of the structure can be changed if I have misinterpreted this figure.)

Response: We have discussed the disulfide linkage between neighboring C855 residues and cited the reference of the different properties between wild-type mouse AE2 and AE2 devoid of TMD cysteine residues in line 93-95.

PIP2 binding site:

All figures with AE1 annotated as (PDB ID: 8CT3) should be accompanied at first mention in each legend by reminders in the corresponding legends that this structure is outward-facing.

Response: Outward-facing AE1 in complex with PIP_2 (PDB ID: 8CT3) we refer to as $AE1_{OF/PIP2}$ were added in the corresponding legends as suggested.

Fig 2a is cited as showing TM7 and HL forming the PIP2 binding site, but this is not interpretable from the figure – it should be annotated with TM7 and HL, or an additional panel (perhaps ribbon or stick) should be added to illustrate the point.

Response: We have annotated Fig. 2a with TM7 and HL as suggested.

Line 97 states that the AE2 PIP2 binding site is consistent with the AE1 PIP2 binding

site, but doesn't mention that AE1 binds adjacent PIP2 and cholesterol molecules, each on one protomer at the interprotomeric cytoplasmic interface. Moreover, Zhang et al (ref 40) find incorporation of cholesteryl hemisuccinate bound in a position close to the cytosolic interprotomeric surface.

Cholesteryl hemisuccinate was present during solubilization in the current study, but appears to be absent from the current AE2 structure.

Can cholesterol be docked into the current AE2 structure or any of the previous structures?

Does the absence or presence of PIP2 or of cholesterol reflect the conditions of solubilization for both AE2 and AE1 in the current work, ref 20 and ref 40?

Response: We have compared densities of cholesterol in AE1 (8CT3) and cholesteryl hemisuccinate in AE2 (8GVH) with corresponding densities in $AE2_{IF/PIP2}$ in Supplementary Fig. 2g. Features of cholesterol and CHS are not as clear as PIP_2 and cholesterol cannot be docked in AE2 at the same position in AE1 (8CT3) by autodock vina and the conditions of solubilization of AE2 is not affected by mutation of residues in PIP_2 binding site during the purification process.

Line 104: This last sentence of p. 4 should be followed by mention of previous AE1 structures in which bound PIP2 was not detected (or not recognized).

Response: Previous AE1 structures determined by Xia et al. were described in line 106-108 and densities showing clear feature of PIP_2 in their map were shown in Supplementary Fig. 2h.

(Methods: lines 255-6: supplemented with how much additional LMNG and CHS?)

Response: 1% LMNG and 0.1% CHS were added in line 284-285.

Mutagenesis of PIP2-interacting residues:

In Fig 3B, the student t -test seems inappropriate – the data should be evaluated by ANOVA with pair-wise post-hoc comparisons to WT. The normalized individual flux values for WT should also be shown. Legend or text should specify absolute value of mean +/-sd WT AE2-mediated Cl--driven base flux. Fig 3Sk and Fig 3Sl should also be evaluated by ANOVA, and not by t-test. Fig. 3Sk is redundant to Fig 3Sl, which provides more meaningful information, rather than a binary determination of surface AE2 positivity or negativity such as shown in Fig. 3Sk.

Response: We have evaluated the data in Fig. 3b and Fig. S3k by ANOVA with pairwise post-hot comparisons to WT, absolute values of mean +/- SEM of wild-type AE2 activity is provided in legend of Fig. 3b and Fig. S3k was removed as suggested.

All experiments of the mutants presented in Fig 3g and Fig 3h should be added as bars to Fig 3b. If numbers don't suffice, n should be increased.

Response: All experiments of mutants have been added as bars to Fig. 3b as suggested.

Authors report but don't discuss the (perhaps unexpected) finding that H1148Y mutation (to the sequence of PIP2-binding AE1) reduces AE2 activity as much as do nonconservative mutations to other PIP2 binding residues.

PIP2 binding is described as "essential to AE2 activity." Indeed, the triple mutant (R932A/H1147A/H1148A) is expressed at the cell surface as well as multiple single PIP2 site mutants, but unlike the 50% reduction in anion exchange activity of the latter, loses all transport activity. And citation of previous mAE2 H1144 mutation may also be consistent with the importance of PIP2 binding. However, at this stage of the text, "essential" is overstatement. The mutatgenesis of these residues later in the text constitutes the required experiment to comment further on the possible function of PIP2 binding.

Line 127: The H1144 mutation result cited "suggests," but does not "indicate," that PIP2 participates in regulation of AE2 activity by pH.

Response: We have changed the word "essential" and "indicated" as suggested. Interaction between PIP_2 and AE1 may be stabilized by protein 4.2 as suggested by Vallese et al (Vallese et al., 2022, PMID:35835865) and we measured the interaction of PIP_2 between various AE2 by autodock4. The estimated free energy of binding of AE2-WT, AE2-R932A, AE2-K1147A, AE2-H1148A and AE2-H1148Y were -7.19 kcal/mol, -6.07 kcal/mol, -3.79 kcal/mol, -6.50 kcal/mol and -6.64 kcal/mol respectively, suggesting the reduced PIP_2 affinity of AE2-H1128Y. However, the mechanism of reduced activity needs to be further studied.

Pp 5-6 concerns comparison of PIP2-bound AE1 structure from ref 20 with the current AE2IF/PIP2 structure. However, inward-facing structures have been published by Zhang et al (ref 40, not mentioned until late in Discussion). These outward- and inward-facing structures must be compared as well, either here or in the next section "Interactions between NTD and of AE2 in absence of PIP2." If current Fig 4a-d is kept, then comparison of current PIP2-bound structure with Zhang et al AE2 structure lacking PIP2 should be added as a supplemental figure, or at least the Gate domain RSMD with that of the published inward-facing Gate domain lacking PIP2 but containing nearby cholesteryl hemisuccinate should be reported. In particular, the interprotomeric subunit interface of ref 40 should be compared to that found in the current work.

Response: We have compared $AE2_{IF/PIP2}$ with inward-facing structures determined by Zhang et al in Supplementary Fig. 4c-e and discussed in line 153-158 as suggested.

Fig. 4b-c legend should specify "one protomer of AE1 TMD (grey) and AE2 TME (colored)……The shift direction of the transmembrane helices from the outward-facing state (of AE1-TMD) to the inward-facing state (of AE2 TMD)….

Fig S5b requires addition of TM annotations.

Response: We have revised the legend of Fig. 4b-c and added TM annotations as suggested.

Fig 4e should add yellow color to uncharged portions of PIP2. DDG's for PIP2 binding

should be compared.

Response: PIP_2 in Fig. 4e has been colored as suggested. The estimated free energy of binding of AE2-WT (-7.19 kcal/mol) is slightly less than the AE2-H1148A (-6.50 kcal/mol) and AE2-H1148Y (-6.64 kcal/mol).

Fig. 4g text treats data as two indistinguishable melting curves. However, the curves (with remarkably tight variance) appear to this reviewer's eye to differ significantly with a ΔTm of ~5oC. Therefore Fig. 4g or related text should include F-test or other statistical comparison of the two melting curves. Such a conclusion would suggest some contribution of conformational change by substitution of charged and polar residues with neutral and smaller Ala.

Response: We agree with the reviewer that mutations may stabilize AE1 proteins. We have included F-test in Fig. 4f and g and revised line 165-170 as suggested.

Fig. 5 legend needs to define AE2IF/REST, since reader encounters the legend prior to text definition on p 7 lines 162-4.

Response: We have defined $AE2_{IF/REST}$ in Fig. 5 legend as suggested.

p. 7 line 165 "inward-facing rotation" should probably be better described as "inward rotational displacement"?

Response: We have replaced the phrase to "inward rotational displacement" as suggested.

Fig 5d legend is incorrect in describing red arrows as indicating the transition from outward-facing to inward-facing state. They rather seem to indicate the computational difference between the IF/PIP2 and IF/REST states of AE2, as noted in text (p.7 lines 164-6).

Response: We have revised Fig. 5d legend as suggested.

p. 7 lines 166-7: Absence of PIP2 from the AE2IF/REST state would be better displayed if the two PIP2 molecules in panel were differentiable by color – perhaps one could have a yellow backbone and the other an orange backbone, with appropriately annotated legends for panels c and d?

Response: We have revised PIP_2 color of $AE2_{IF/PIP2}$ in Fig. 5c and 5d with annotated legends as suggested.

Fig. 5e should add annotation of the mutated residues 932, 1147 and 1148 in both "AE2IF/REST and AE2IF/PIP2 chains.

Response: We have added annotation of the mutated residues as suggested.

Fig 5f legend should specify that the partial structure shown is from (and visible only in) AE2IF/REST.

Response: We have specified the panel and legends as suggested.

Fig 5f should be modified to indicate the specific charge-pairs, and at least some of the proposed hydrophobic interactions between TMD loop 10-11 and stabilized NTD, as described in lines 172-5. Same should be done in Fig. 5g. These interactions should be directly compared to the TMD-NTD interface revealed in Fig 2 of ref. 40.

Response: We have modified Fig. 5f-g and compared with Fig 2 of ref. 40 in Supplementary Fig. 6d-e as suggested.

Mutation of the NTD region shown at right in Fig 5f acid shifts the pH vs. activity curve (increases activity over a range of pH values) whereas further deletion of the downstream NTD region shown in Fig 5g can rescue normal pH-sensitive activity. How does these earlier findings relate to the current results?

Response: Previous study showed that deletion of 1-328 residues at N-terminal of mAE2 (corresponding to 1-332 of hAE2) nearly abolished the activating effect of butyrate removal(Stewart et al., 2001, PMID:11546673). $AE2_{IF/REST}$ structure suggested that deletion 320-332 (colored in blue) may disrupt the interaction of Nβ1 (325-332) and Nβ2 (338-346) and further destabilize the NTD region (colored in yellow) shown in Fig. 5f-5g and partially explained the NBD regulatory mechanism of intracellular pH to AE2 activity.

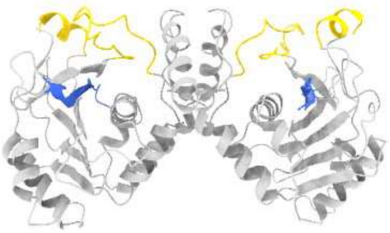

NTD regions in Fig. 5f interact with TMD through loop between TM10 and TM11. Deletion of 1-347 residues of mAE2 (1-351 residues of hAE2) and mutations in loop between TM10 and TM11 have similar effect on the regulation of AE2 by extracellular pH, suggesting interaction shown in Fig. 5f is involved in the regulation (Stewart et al., 2001, PMID:11546673; Stewart et al., 2009, PMID:19103596). $AE2_{IF/REST}$ state is supposed to be a resting state. Disruption of interaction between TMD and NTD probably made AE2 more easily to be activated by alkaline extracellular pH. We can only hypothesize that further deletion of 348-391 residues of mAE2 improved the interaction between 506-511 residues of NTD and loop between TM10 and TMD11 (Fig. 5f), as we do not have evidence currently.

Lines 185-6. "…even on the SLC4 family transporters" sounds inappropriately hypothetical, as PIP2 has already been published as an activity regulator of SLC4A4 and SLC4A7 variants. Please update and revise. Ref 36 recognizing this for SLC4A4 is currently not mentioned until Discussion.

Response: We have modified the phrase and cite ref of PIP_2 regulation on SLC4 family proteins in line 206-208 as suggested.

Fig 6c should remove the yellow PIP2 circle entirely for clarity -or perhaps change its color to grey.

Response: We have removed the yellow PIP2 circle as suggested.

Discussion:

Lines 195-8 claim that PIP2 disrupts the interaction between TMD and NTD, preventing inhibition by NTD of AE2 conformational transition and promoting substrate transport. This is overstated. The study does show that a triple mutant that disrupts PIP2 binding stabilizes the TMD-NTD interaction and is inactive as a transporter. That experiment indeed suggests, but does not prove, that PIP2 binding is required for anion transport. However, direct functional evidence of NTD inhibitor interaction with TMD is not demonstrated here. Authors should test the effects of PIP2 depletion vs supplementation on activity of wildtype AE2 in intact cells or in proteoliposomes of defined lipid compositions.

Response: We have tested the effects of PIP_2 depletion as suggested. Cells transfected with wildtype AE2 were incubated with phosphoinositide 4-kinases inhibitor wortmannin or PLC activator m-3m3FBS for 30 min before measurements. Results show that either way of PIP_2 depletion reduced the activity of wildtype AE2 in intact cells (data are shown as mean ± sem).

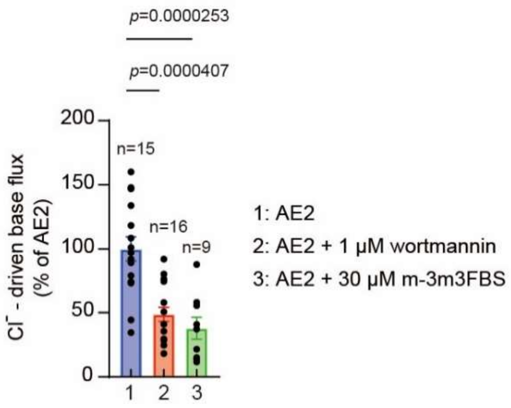

Authors should summarize previous work on regulation of red cell AE1 activity by cholesterol depletion and supplementation. The authors should also link the current work to previous biophysical studies comparing the effects of cholesterol and PIP2 on AE1 (+/- glycophorin A) in lipid bilayers.

Response: We have summarized previous work on regulation of AE1 by cholesterol depletion and supplementation and linked our work with previous biophysical studies in line 213-216.

Not addressed at all in the paper is the difference between the currently proposed regulation of activity by PIP2, and the previously proposed regulation of activity by insertion of the AE2 C-terminal tail into the inner vestibule detected in the low pH inward-facing structure (ref 40).

Response: We have discussed the differences between PIP_2 and AE2 C-terminal tail in line 253-257 as suggested.

Can PIP2 be detected or be docked into any of the reported AE2 structures of ref. 40? The Fig 6 model is unclear about the meaning of the green tail of the Core domain. Is this tail the TMD C-terminus? Or is it the section of the TM10-11 cytoplasmic loop that is stabilized by PIP2? If the former, then the role of the C-terminus is incorporated into the Fig. 6 model without any evidence in the current paper and without clear attribution to ref 40.

How can PIP2 be essential for AE2 function when AE2 functions in the apparent absence of PIP2 in ref 40? Was PIP2 present but undetected in some of the structures of ref 40? Were detergent solubilization conditions and imaging conditions sufficiently different to account for the absence of presence of PIP2? The Fig 6a schematic with PIP2 at the interprotomeric interface shows each protomer's inner vestibule apparently blocked by its own C-terminal peptide – was such a structure present among the image class-averages of cryo-EM particles, but not reported here?

Response: PIP_2 can be recognized in the map of $AE2_{out}^{basic\text{-}KNO3}$ and $AE2^{NaCl}$ of (Zhang et al., 2023, PMID:37002221) (Supplementary Fig. 6g and 6f). Tail of core domain in Fig. 6a-b represents the loop between TM10 and TM11, which is stabilized by the absence of PIP_2. Dashed line indicates that loop is flexible at this state. We have revised the legend and discussion in line 233-239.

Do PIP2 and the C-terminal peptide compete for the same binding site in the inner vestibule? Do they exert the same or different effects on transport activity?

Does inter-protomeric PIP2 characterize only the inward state of AE2? Could PIP2 be unrelated or secondarily related to the outward-inward transition, rather controlling anion unbinding from the inward-facing AE2 under conditions permitting release to the cytoplasm?

Response: PIP_2 binds at dimer interface as activator and C-terminal peptide binds in the inner vestibule as inhibitor. PIP_2 can be identified in both inward- and outward-facing states of AE2 (Supplementary Fig. 6g and 6f) suggesting the involvement in outward-inward transition.

Methods:

Line 265: Define buffer and salt composition of concentrated AE2 sample deposited on grid for cryo-EM.

Line 277: Define CTF as Contrast Transfer Function

Response: We have defined the buffer and CTF in Line294-295 and line307.

Line 285 "as previously described" – cite ref or restate as "as described above"

Line 295: clarify that cells for flow cytometry were transfected with HA-tagged AE2 carrying C-terminal GFP, if that was the case.

Response: We have restated as “as described above” and clarified the tag of AE2 for flow cytometry as suggested.

Statistics: Line 325 mentions standard error, but all legends mention standard deviation. Since v

Response: We have changed the word as suggested.

Minor points

English syntax and grammar need correction throughout manuscript

Throughout manuscript, abbreviations are introduced without definition. This should be remedied.

Response: We have remedied the syntax, grammar and abbreviations throughout manuscript as suggested.

Fig S1 legend: a) “motion-corrected”; c) provide some detail describing blue, gray and yellow low-res structures and meaning of the orange box; f-h: provide descriptions of the three views shown, and explanation of the lower red (low res) structures at the bottom of panels f and g

Response: We have revised Fig. S1 legend as suggested.

Fig 1 legend: a and b) define gray lipids. d) define residue # at N-terminus of Nβ1. Define residue #s at C-terminus of resolved region beyond NH8 and N-terminus of resolved region preceding H1.

Response: We have defined grey lipids and included detailed information of model content in the revised Supplementary Table 1.

Fig. S2d legend or relevant text should list the interacting inter-protomeric residue pairs. Fig S2d shows an inter-protomeric disulfide linkage between C855 of each protomer, but this is not mentioned in the relevant text on p 4 lines 89-90. Fig S2e legend should specify that image of putative chloride binding site is from an inward-facing form, since the coordination site was accessible from the cytoplasmic side, as described in text.

Response: We have listed the interacting residue pairs, mentioned C855 in line 93-95 and specified the putative chloride binding site is from $AE2_{IF/PIP2}$.

Fig 2b should show the AE2 interacting residue mesh in blue rather than gray, which is hard to see adjacent to the yellow mesh, even enlarged 4-fold.

Response: We have changed the color to blue as suggested.

Figs S3k and S3l need X Axis labels either in place of or to supplement the current color keys, which are difficult to read. The colored circles must be larger, in addition to labeling the columns along the X axis using angled labels.

Response: We have and modified Fig. s3 as suggested

Reviewer #2 (Remarks to the Author):

This is an impressive cryo-EM study that reports on the 3.2 A structure of PIP2 binding to the inward-facing configuration of partial AE2 (TMD). The authors extend their analysis and description of PIP2 binding by also solving the structure of AE2 unbound to PIP2, and the 2 structures provide insight into the role of the N terminal domain (NTD) and associated structural changes. Further support for the proposed structures and models is provided my mutagenesis data of the PIP2-binding site highlighting its impact on function, and comparative analysis to recent work by Vallese et al. identifying PIP2 binding to AE1. This in an important study that provides insight into a regulatory mechanism of a membrane acid-base transporter, and the findings will help the field understand such regulation of other related bicarbonate transporters, and how it may link to their pH sensing mechanism(s). A number of issues need to be addressed.

We thank this reviewer for the highly constructive and helpful comments.

1. The current emphasis on PIP2 binding/regulation being linked to the protein's pH sensor is too strong with the limited data. pH sensing is not examined in the current study. This aspect needs to be toned down in the abstract and elsewhere.

Response: We have made more comparison of $AE2_{IF/REST}$ and AE2 in acidic pH and changed the emphasis to PIP_2 regulation of AE2 conformation and activity rather than pH sensing as suggested.

2. There is discussion on p. 4 about the "flexibility" and possible "unstable" nature of NTD in the structural analysis. How can the authors then be confident in their assessment of the PIP2-sensitive positioning of the NTD in their model proposed in Fig. 6.

Response: Position of NTD in Fig. 6c is determined by $AE2_{IF/REST}$ structure and position of flexible NTD in Fig. 6a and 6b which cannot be defined were indicated by dashed lines.

3. Fig. 3 functional analysis. Based on the associated localization data (Suppl. Fig. 3), these mutations have effects on both localization and/or function. When factoring in a decrease in expression for R932 and H1148 mutations, for example, there appears to be a decrease in expression by ~25%, so the subsequent functional decrease of ~25% appears quite mild. This needs to be made clear and discussed.

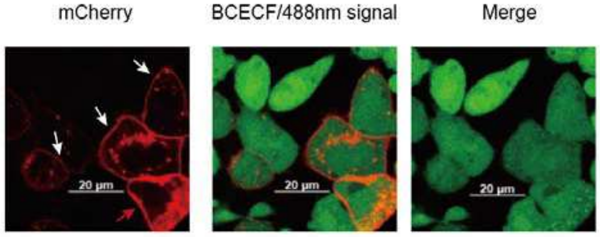

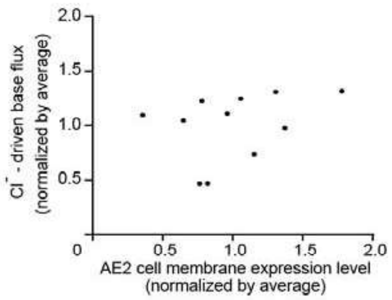

Response: AE2 expressing cells selected (good cells indicated by white arrows) for the activity measurements were without large accumulation of AE2 (bad cell indicated by red arrow) in organelles. Activities were not significantly affected by the fluorescence intensity of plasma membranes shown in figure above. This might be due to the overexpression of AE2 or the total amount of PIP_2 is limited on the plasma membranes.

Also, the Fig. 3b analysis doesn't take into account the pHi sensitivity of the measurements. For example, the re-addition of Cl- in experiments g and h may have had no acidifying effect because the pHi was already low. This needs to be discussed. In theory, the pHi values could be low because of reduced activity of the AE2 to begin with. However, based to the previous paragraph, would a 50% decrease in activity/expression account for this? It might be worth looking at the resting pHi of each of these mutants to help in proposing some explanations.

In light of these points above, it seems premature to conclude that any of these mutations "disrupted the substrate coordination site"

Response: We have discussed the phenomenon that intracellular pH of cells transfected with mutant AE2 rundown quickly during equilibrating with buffer without Cl^- in line 124-125 and corrected the methods of activity measurements. We have measured the activity of wild-type AE2 and AE2 with R932/K1147/H1148A mutation with shorter equilibrating time. Intracellular pH of cell transfected with mutant AE2 rundown quickly and the speed of acidification hardly changed with the addition of Cl^-. We have replaced Fig. 3g and 3h with relatively stable traces started at higher pHi for better interpretation.

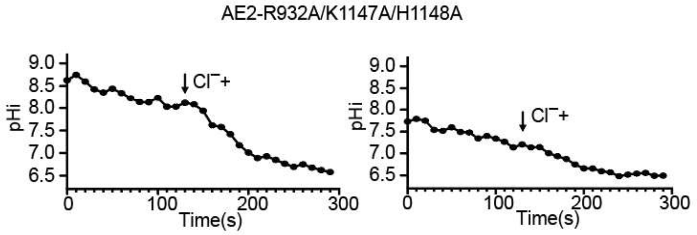

4. It is not clear why the authors conclude that the difference in traces in Fig. 4g doesn't represent a thermal shift.

Response: We agree with the reviewer that the traces are different. Mutations of AE1 stabilized the proteins so we did not conclude that PIP_2 regulation activity by stabilizing AE2 dimerization. We have revised line 165-167 for better interpretation.

5. Overall, this is a well-written and logically presented story. However, grammar needs to be improved in many places.

Response: We have remedied the grammar throughout manuscript as suggested.

Reviewer #3 (Remarks to the Author):

In the manuscript "Structural insights into the lipid regulation of human anion exchanger 2" Zhang et al determine cryoEM structures of AE2 and identify a PIP2 binding site that is important for transport function. Through structural and functional analyses, Zhang et al show that disruption of the PIP2 binding site causes conformational rearrangements within the TMD that promote association with the NTD. Based on their work the authors propose that PIP2 allosterically activates AE2 for substrate transport. The presented work complements previous structural studies on AE2, and provides much needed structural insight into the modulation of transporters by membrane components such as PIP2. The presented work is interesting, but a confusing discussion/summary cartoon, lack of comparison with previous work, and incomplete modeling/interpretation of the PIP2 site dampens enthusiasm.

We thank this reviewer for the thorough evaluation of our structures and the highly constructive and helpful comments.

Several major points need to be addressed:

• The cartoon shown in Figure 6 is confusing and not very helpful to understand the role of PIP2 in modulation of transporter activity: The NTD is outlined with dashed lines in all panels. The difference between bound and unbound PIP2 is only indicated by solid and dashed lines rather than removing the molecule entirely. The legend states "In the presence of PIP2, substrates binding sites is accessible to Cl- in the outward-facing conformation and to HCO3- in the inward facing conformation", but the authors provide a Chloride- and PIP2-bound IF state structure. So none of the states proposed here are backed up by structures in this manuscript. The authors also discuss a pH-sensing role of PIP2, which is missing from this cartoon. Overall, this figure is important to understanding the findings reported herein and needs to be much improved.

Response: We have modified Fig. 6 and PIP_2 density in the outward-facing map of AE2 bound with $HCO3^-$ is shown in Fig. S6f. We have discussed and compared our structures with AE2 determined in acidic and alkaline pH in Fig. S5c-d and S6b-e and in line 187-191 as suggested.

• The density for the modeled PIP2 atoms is excellent, but the PIP2 molecule is not complete – much of the acyl chain is missing. The authors need to state that in the manuscript, as it is currently implied that the whole molecule was built. Also, the cryoEM density shows that there are other membrane components involved in modulating AE2 (and perhaps PIP2) activity, but PIP2 is the only modeled lipid. Given the focus on lipid modulation in this work, some of these should at least be modeled, particularly as previous work identifies many lipids and sterols in the modulation of SLC4 transporters and AE2. There are two additional lipid-like molecules in the immediate vicinity of PIP2 that are likely the most relevant to the subject of the paper. One of them directly packs against the acyl-chain of PIP2 and likely directly impacts PIP2 affinity and function. Previous work of ion channels (e.g. PMID: 34433062 for ion channels) has structurally elucidated how sterols contribute to PIP2 binding.

Something similar needs to be done here to define the PIP2 pocket in detail.

Response: We have stated that PIP_2 molecules are not complete in line 97-98 as suggested. We have color the lipid-like density corresponding to cholesterol in AE1 (Vallese et al., 2022, PMID:35835865) and cholesteryl hemisuccinate in AE2 (Zhang et al., 2023, PMID:37002221) in red and pink in Fig. S2g. Our current evidences of PIP_2 regulation were mainly focus on the inositol head of the molecule. However, different from PIP_2, cholesterol only show clear features in AE1 determined by Vallese et al. and cholesteryl hemisuccinate can only be identified in AE2 structure determined at acidic pH. Therefore, we cannot conclude whether cholesterol regulates the binding of PIP_2.

• Related to the previous point, the PIP2 density figure (Supplementary Figure 2f) needs to show density for the nearby residues of the binding pocket to provide an assessment of model quality of the sidechains involved. Within this context, one should also show the neighboring lipid/sterol density that plays a role in coordinating PIP2.

Response: We have modified Fig. 2b-c and provided Fig. S2g for better visualization of PIP_2 binding pocket and neighboring lipid-like densities.

• Previous work reported six AE2 structures, and this work should already be cited in the introduction in line 62, and not just later. Despite these structures, the authors do not perform any structural comparison with the previous AE2 states, which could have provided novel insights and validated the author's work. For instance, overlay with 8GV9 shows a very different positioning of the Chloride ion compared to the herein presented TMD structure. Since the herein reported Chloride ion is more than 4.5 A from R1060 and does not agree with previously reported work (Zhekova et al, Capper et al), this could also be water, other buffer components, or noise from the map. At the very least, the authors would have to explain the discrepancy to previous work. Similarly, the manuscript could benefit from comparison to other structural AE2 states. For instance, what does the PIP2 binding site look like in the OF state? How do basic or acidic conditions change the conformation of the PIP2 binding site? These analyses would greatly strengthen this work.

Response: We have cited the ref in the introduction and compared the structures of AE2 in different states as suggested. Position of the chloride were corrected. PIP_2 also binds at the dimer interface in the OF state (Supplementary Fig. 6f). Mutations in PIP_2 binding site and acidic pH change the conformation of TM7 and acidic pH further causes insertion of the C-terminal loop into the inner vestibule of AE2. We have discussed these in line 252-257 as suggested.

• There are several peculiar details in the flux data. While overall consistent with the author's hypothesis, only WT AE2 displays a stable pH baseline before addition of chloride. For instance, cellular pH of the R932A mutant drops by 0.4 units before the authors even add Chloride. That raises several questions, did the authors not provide sufficient time to equilibrate the system, or do the mutant cause ion leakage? Why do the recordings of the triple mutant and the R1060A mutant start at pH 6.9 compared to

the other experiments that start at cellular pH? I would also recommend to expand the alignment in Figure 3i to include the complete family of SLC4 transporters as the authors later make a sweeping statement about PIP2 pockets being potentially present in all SLC4 transporters.

Response: The system was equilibrated more than 5 min before addition of chloride. Intracellular pH of cells transfected with mutant AE2 tended to be stable at relatively low pHi, compared with wild-type AE2. We have measured the activity of wild-type AE2 and AE2 with R932/K1147/H1148A mutation with shorter balancing time and added chloride at relatively alkaline pH, however, the speed of acidification hardly changed by the addition of Cl^-. We have replaced Fig. 3g and 3h with relatively stable traces started at higher pHi for better interpretation. We have expanded the alignment in Fig. 3i to include the complete family of SLC4 transporters as suggested.

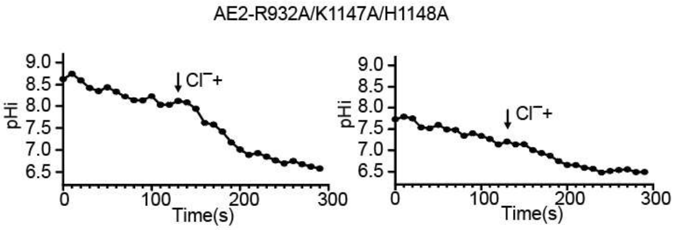

• There are numerous typos and grammatical errors throughout the manuscript, and several statements are awkwardly phrased. While there are too many to list all of them, here are some that need clarification:

o Line 46/47: "Alternative promoters of the SLC4a2 gene give rise to three AE2 isoforms…" It sounds like three different promoters can produce 3 different isoforms from the same coding sequence.

Response: We have changed the word to splicing as suggested in line 46.

o Line 76: "SEC" is not defined

Response: We have defined SEC in line 78-79 as suggested.

o Line 84/85: "..suggesting that [the] NTD was unstable.." This implies that the NTD unfolds or aggregates. I suppose the authors want to state that the NTD was too flexible to assume a conformation detectable by cryoEM.

Response: We have revised the sentence as suggested.

o Line 120/121: "Phylogenetic analysis revealed that residues consist of the PIP2 binding site are absolutely conserve among AE2 orthologs…" There is no phylogenetic analysis in this manuscript, only sequence alignment of the PIP2 pocket. "absolutely" needs to be removed, and several typos need to be fixed in this section.

Response: We have revised line 132-134 as suggested.

o Line 136/137: "[The] core domain of AE2IF/PIP2 rotates towards the gate and

domain and [shifts] downward to the cytosolic side …”

Response: We have corrected the errors in line 147-148.

o Line 176/177: “Ankyrin complexes solved by others display extensive interfaces between AE1 and protein 4.2 indicating a strong relationship between these proteins.” “relationship” is awkward in this context. Rephrase!

Response: We have revised the sentence as suggested in line198-199.

O Line 188: “Although PIP2 is a minority phospholipid…” “Minority” is awkward in this context, and it doesn’t matter that it isn’t the main constituent of the membrane. Remove!

Response: We have removed the word as suggested in line 210.

o Line 202/203: “mutating R932, K1147 and H1148 has (have) little impact on the structural stability and plasma membrane trafficking.” Supplementary figure 31 shows that the triple mutant reduces membrane expression by ~30%, so this statement needs to be corrected.

Response: We have corrected the statement in line 225-226 as suggested.

References:

Stewart, A.K., Chernova, M.N., Kunes, Y.Z., and Alper, S.L. (2001). Regulation of AE2 anion exchanger by intracellular pH: critical regions of the NH(2)-terminal cytoplasmic domain. Am J Physiol Cell Physiol *281*, C1344-1354.

Stewart, A.K., Kurschat, C.E., Vaughan-Jones, R.D., and Alper, S.L. (2009). Putative re-entrant loop 1 of AE2 transmembrane domain has a major role in acute regulation of anion exchange by pH. J Biol Chem *284*, 6126-6139.

Vallese, F., Kim, K., Yen, L.Y., Johnston, J.D., Noble, A.J., Cali, T., and Clarke, O.B. (2022). Architecture of the human erythrocyte ankyrin-1 complex. Nat Struct Mol Biol *29*, 706-718.

Zhang, Q., Jian, L., Yao, D., Rao, B., Xia, Y., Hu, K., Li, S., Shen, Y., Cao, M., Qin, A., *et al.* (2023). The structural basis of the pH-homeostasis mediated by the Cl(-)/HCO(3)(-) exchanger, AE2. Nat Commun *14*, 1812.

REVIEWER COMMENTS

Reviewer #1 (Remarks to the Author):

Major points

General: Grammar and syntax still problematic throughout; language sometimes confusing.

How do authors reconcile that while triple mutation of the hAE2 PIP2 binding residues does not alter temperature denaturation curve of the holoprotein, NTD and apparently also holoprotein is/are nonetheless rigidified by the triple mutation. The ability to resolve the non-extended NTD structure as part of the structure of the triple mutant lacking bound PIP2 suggests remarkable stabilization of the holoprotein. Why don't the two types of data better concur, as in the case of hAE1? Might the disulfide at C855 have anything to do with this difference? How confident are the authors that the PIP2-unbound inward structure lacking activity fails to bind anions anywhere along the anion translocation pathway (as in the Fig 6 cartoon), since the anion substrate were suboptimally resolved?

Please add to Methods a description of and citation for the thermal stability assay, currently lacking. At what pH values were the hAE1 and hAE2 thermal stability assays conducted? In what buffer composition, including which substrate(s)? Were pH-dependent thermal stability assays conducted? How was densitometry standardized for the thermal stability results? Did hAE! and hAE2 differ in the proportion of SDS-resistant dimer? Were both monomer and dimer summed for the thermal stability analysis? Or was only monomer quantitated?

Line 125: "rundown" is vague here – describe more precisely.

126: Cells presented in Fig 3D and 3F are not at steady state at time of reintroduction of chloride. Fig 3F steady-state is questionable. These must be considered unreliable data.

130: How does R1060A disrupt the substrate coordination site? Fig 3H shows only loss-of-function. This statement needs supporting citation or revision.

153-158: Mention whether pink CHS (SFig. 4d) corresponds to one of the poorly-defined putative lipid densities of the current hAE2 structure.

161: As noted above, Fig. 3g is unacceptable as is. Baseline pre-chloride pHi must be stable before bath change.

165-167: Text states that AE1 triple mutant thermal stability "was not reduced," but the F-test comparison between blue and red curves in Fig 4G is highly significant as reported in figure 4g legend. Text must be modified to emphasize that based on thermal stability measurements, mutation of the PIP2 binding site appears to destabilize hAE1, but does has no detectable effect on hAE2 stability.

189-190: Change “intracellular pH” to “acidic intracellular pH”

Fig. 6 Ctail of Core should be labeled or described in legend.

Discussion

223-228: Conclusions about role of PIP2 binding in anion exchange activity require improvement of the transport data of Fig. 3 discussed above.

236-237: Where do the authors show that the binding pocket of PIP2 is “disrupted by acidic intracellular pH? “ Please show or refer to the data (perhaps PDB ID:8GVH)? Or perhaps change “disrupted” to “conformationally altered so as to lose or alter PIP2 binding.

Remaining minor points

Lines 86-88: change order of sentence to present structural findings first, and conclusions afterwards.

98: change “uncomplete” to “incompletely visualized and defined”.

102: define IL4 and IL7 as “?intracellular loops”?

105: change “lack of features” to “poor resolution” or an equivalent.

106: change “could also be” to “were”.

107-8: change “blobs” to something more informative, and clarify that the “blobs” refer to the current structure rather than to the ref. 22 structure.

108: Refer to Fig 1 to help readers orient themselves to the locations of HL and H5 here and in Fig 2A legend.

120-130 and throughout: Establish consistent use either of one letter or three letter AA code.

125: change “consisting” to “comprising” or “contributing to”

175: Change “circumstance” to “conditions”

190-191: Please color the backbone chains of NTD in SFigs 6D,6E differently from the TMD backbones, as done for Figs 5F and 5G.

Discussion:

231-235: Add citation for Zhang et al.

Reviewer #2 (Remarks to the Author):

The authors have been responsive to the Reviewers' comments. However, additional clarity and information is needed.

1. The grammar in many places still makes the ms difficult to read and understand fully. This still needs to be addressed.

2. The authors should be using SD, not SEM.

3. More conventional use reporting P values is needed; the excessive accuracy is not necessary.

4. The experiment provided to the Rs about PIP2 depletion decreasing AE2 activity should be included in the ms, or at least mentioned as unpublished.

5. Fig. 3 data. I am still concerned that this section doesn't appropriately make clear to the reader that some of these decreases in activity are due in part to reduced plasma-membrane expression. This should be clarified with more detail provided.

6. "AE2 rundown quickly" should be rewritten, and I'm not sure about "equilibrating." You might refer to a drifting pHi baseline or a decreasing pHi that either reaches or does not reach a resting pHi. In light of the new experiments at higher pHi compared to previously shown experiments, I think it's important to mention that for specific mutants, the rate of pHi decrease with Cl- readdition is similar for a range of pHi values in these experiments (and give that range).

7. Previous studies of PIP2 regulation of NBCe1...Check that literature because they were not all patch-clamp studies.

Reviewer #3 (Remarks to the Author):

The revised manuscript by Zhang et al is much improved and most of my suggestions and comments have been addressed. However, the manuscript remains challenging to read due to a large number of typos as well as grammar and syntax errors – many of which have been introduced during the revision. I therefore urgently recommend vigorous proofreading of the manuscript, maybe even by a third-party service provider.

Point-by-point response to reviewers

We are grateful for the thorough evaluation of our manuscript from the three reviewers. A detailed point-by-point response to all comments, suggestions and requests of the reviewers are provided in this document.

Reviewer #1 (Remarks to the Author):

Major points

General: Grammar and syntax still problematic throughout; language sometimes confusing.

Response: We have proofread the manuscript comprehensively and payed particular attention to improving grammar and syntax as requested.

How do authors reconcile that while triple mutation of the hAE2 PIP2 binding residues does not alter temperature denaturation curve of the holoprotein, NTD and apparently also holoprotein is/are nonetheless rigidified by the triple mutation. The ability to resolve the non-extended NTD structure as part of the structure of the triple mutant lacking bound PIP2 suggests remarkable stabilization of the holoprotein. Why don't the two types of data better concur, as in the case of hAE1? Might the disulfide at C855 have anything to do with this difference? How confident are the authors that the PIP2-unbound inward structure lacking activity fails to bind anions anywhere along the anion translocation pathway (as in the Fig 6 cartoon), since the anion substrate were suboptimally resolved?

Response: We have tested the effect of mutating C855 on the thermal stability of AE2 as suggested. Our results (below) show that the inability to form a disulfide bond between C855 residues does not have an effect on the thermal stability of AE2 as the triple mutation does on AE1.

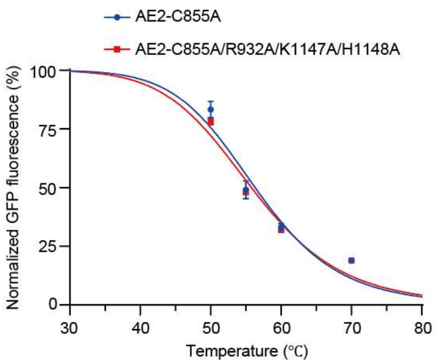

Fig. 6a-b are presented to describe conformational changes during the substrate transport process whereas the data in Fig. 6c describes the conformation of AE2 in the

resting state. Substrates cannot be transported by AE2 in the resting state as described in our model. We do not yet have any evidence indicating whether or not anions could bind to the substrate binding pocket of AE2 in its resting state.

Please add to Methods a description of and citation for the thermal stability assay, currently lacking. At what pH values were the hAE1 and hAE2 thermal stability assays conducted? In what buffer composition, including which substrate(s)? Were pH-dependent thermal stability assays conducted? How was densitometry standardized for the thermal stability results? Did hAE! and hAE2 differ in the proportion of SDS-resistant dimer? Were both monomer and dimer summed for the thermal stability analysis? Or was only monomer quantitated?

Response: We have added the methods and citation for the thermal stability assay in lines 369-378 of the revised manuscript as requested. Assays were conducted in buffer containing 150 mM NaCl, 1% LMNG, 0.1% CHS and 20 mM HEPES at pH 7.4. The fluorescence of residual proteins was detected by fluorescence size exclusion chromatography. Peak heights of the AEs dimer were used for the analysis. Assays of pH-dependent thermal stability of AEs were conducted as suggested. These experiments showed that buffer pH influences AE2 more than AE1 and that AE2 is more stable at acidic pH (pH 6.5).

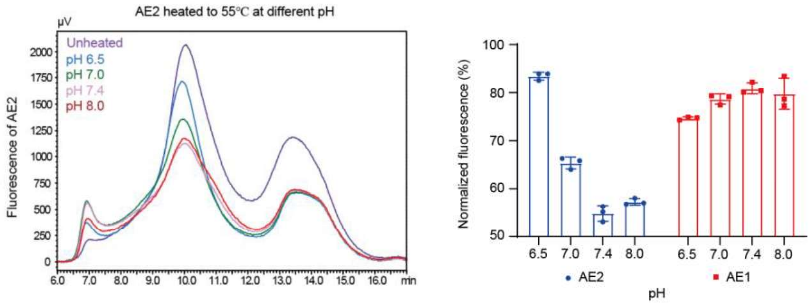

The proportion of dimerized GFP-tagged AE1 and AE2 in cell lysates was measured by GFP fluorescence in SDS-PAGE. These experiments showed that the proportion of dimerized AE2 is greater than that for AE1 almost certainly due to the disulfide bond formed between C855 residues in the protomers (white and red arrows below indicating AE dimers and monomers, respectively).

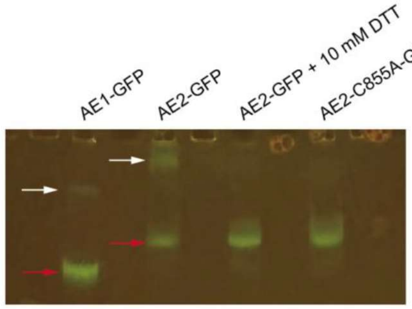

Line 125: “rundown” is vague here – describe more precisely.

Response: We have changed “rundown” to “stabilized at more acidic intracellular pH than cells transfected with wild-type AE2” in line 129.

126: Cells presented in Fig 3D and 3F are not at steady state at time of reintroduction of chloride. Fig 3F steady-state is questionable. These must be considered unreliable data.

Response: We have conducted more pHi measurements to obtain baselines with considerably less drift before the addition of chloride. Data obtained with $[H^+]$ changing rates <1% of the wild-type AE2 activity before the addition of chloride are considered reliable . Fig. 3b-h were replaced with this more reliable data .

130: How does R1060A disrupt the substrate coordination site? Fig 3H shows only loss-of-function. This statement needs supporting citation or revision.

Response: $AE2_{IF/PIP2}$ of ours and $hAE2^{NaCl}$ determined by (Zhang et al., 2023, PMID:37002221) showed Cl^- ion located near R1060 and R1060 is the only positively charged residues in the substrate binding pocket. We have cited the reference as requested in line 134.

153-158: Mention whether pink CHS (SFig. 4d) corresponds to one of the poorly-defined putative lipid densities of the current hAE2 structure.

Response: A description of the pink CHS in Supplementary Fig. 4d has been added in the legend as suggested (lines 721). Pink CHS belongs to 8GVH and corresponds to the pink lipid-like density of $AE2_{IF/PIP2}$ as indicated in Supplementary Fig. 2g.

161: As noted above, Fig. 3g is unacceptable as is. Baseline pre-chloride pHi must be stable before bath change.

Response: Traces with baselines with excessive drift before the addition of chloride have been excluded as suggested and Fig. 3g has been replaced with more rigorously obtained data (minimal baseline drift).

165-167: Text states that AE1 triple mutant thermal stability “was not reduced,” but the F-test comparison between blue and red curves in Fig 4G is highly significant as reported in figure 4g legend. Text must be modified to emphasize that based on thermal stability measurements, mutation of the PIP2 binding site appears to destabilize hAE1, but does has no detectable effect on hAE2 stability.

Response: We agree with the reviewer that the thermal stability of wild-type and triple mutant AE1 is different and triple mutations stabilized AE1. We have changed “was not

reduced" to "increased" and have added the Tm_{50} of wild-type and mutant AE1 to better illustrate this finding in lines 174-175.

189-190: Change "intracellular pH" to "acidic intracellular pH"

Response: Words have been changed as requested in lines 200-201.

Fig. 6 Ctail of Core should be labeled or described in legend.

Response: The tail of the core domain shown in Fig. 6 represents the intracellular loop between TM10 and TM11. In the revised manuscript, we have added this information to the legend of Fig. 6 to better describe the figure in lines 661-664.

Discussion

223-228: Conclusions about role of PIP2 binding in anion exchange activity require improvement of the transport data of Fig. 3 discussed above.

Response: Traces with drifting baseline before addition of chloride have been excluded from analysis and the data in Fig. 3 have been replaced with data from experiments with more stable baselines as requested.

236-237: Where do the authors show that the binding pocket of PIP2 is "disrupted by acidic intracellular pH? " Please show or refer to the data (perhaps PDB ID:8GVH)? Or perhaps change "disrupted" to "conformationally altered so as to lose or alter PIP2 binding.

Response: We have changed "disrupted" to "conformationally altered" as suggested. Line 251.

Remaining minor points

Lines 86-88: change order of sentence to present structural findings first, and conclusions afterwards.

Response: The order of information presented has been changed as suggested. Lines 87-90.

98: change "uncomplete" to "incompletely visualized and defined".

Response: Words have been changed as requested. Line 100.

102: define IL4 and IL7 as "?intracellular loops"?

Response: IL has been defined as the abbreviation for 'intracellular loop' as requested. Line 104.

105: change “lack of features” to “poor resolution” or an equivalent.
106: change “could also be” to “were”.

Response: Changes to the text have been made as requested. Lines 108-109.

107-8: change “blobs” to something more informative, and clarify that the “blobs” refer to the current structure rather than to the ref. 22 structure.

Response: We have replaced ‘blobs’ with ‘density’ as requested and made clear that the reference is to the density of AE1 (EMD-26142) resolved by (Xia et al., 2022, PMID:35655099). Lines 108-110.

108: Refer to Fig 1 to help readers orient themselves to the locations of HL and H5 here and in Fig 2A legend.

Response: As requested, we have referred to Fig. 1 in lines 116-117 and in the legend of Fig. 2a.

120-130 and throughout: Establish consistent use either of one letter or three letter AA code.

Response: In the revised manuscript, we now use the one letter amino acid code throughout as requested.

125: change “consisting” to “comprising” or “contributing to”
175: Change “circumstance” to “conditions”

Response: These changes have been made as requested. Lines 126-129 have been rewritten in lines 130-133. “Circumstance” in line 175 has been changed in line 185.

190-191: Please color the backbone chains of NTD in SFigs 6D,6E differently from the TMD backbones, as done for Figs 5F and 5G.

Response: Backbone chains of the NTDs in Supplementary Fig 6d and 6e have been re-colored as requested.

Discussion:
231-235: Add citation for Zhang et al.

Response: We have added this citation in line 251 as requested.

Reviewer #2 (Remarks to the Author):

The authors have been responsive to the Reviewers' comments. However, additional clarity and information is needed.

1. The grammar in many places still makes the ms difficult to read and understand fully. This still needs to be addressed.

Response: See response to Reviewer #1. We have proofread and revised the manuscript comprehensively to improve grammar and syntax as requested.

2. The authors should be using SD, not SEM.

Response: We have changed SEM in Fig. 3b, Fig. 4f-g and Supplementary Fig. 3k to SD as requested.

3. More conventional use reporting P values is needed; the excessive accuracy is not necessary.

Response: P values in Fig. 3b and Supplementary Fig 3k are reported using conventional way as suggested (*$p < 0.05$, **$p < 0.01$, ***$p < 0.001$).

4. The experiment provided to the Rs about PIP2 depletion decreasing AE2 activity should be included in the ms, or at least mentioned as unpublished.

Response: We have added the results of the PIP_2 depletion experiments in Fig. 3j and lines 134-135 as requested.

5. Fig. 3 data. I am still concerned that this section doesn't appropriately make clear to the reader that some of these decreases in activity are due in part to reduced plasma-membrane expression. This should be clarified with more detail provided.

Response: We have discussed the effect of the mutations in lines 126-134 of the revised manuscript as suggested to indicate that decreased membrane localization and Cl^--driven base efflux both compromise the pH regulating ability of AE2. Additional details have been provided in the legends of Supplementary Fig. 3k.

6. "AE2 rundown quickly" should be rewritten, and I'm not sure about "equilibrating." You might refer to a drifting pHi baseline or a decreasing pHi that either reaches or does not reach a resting pHi. In light of the new experiments at higher pHi compared to previously shown experiments, I think it's important to mention that for specific mutants, the rate of pHi decrease with Cl- readdition is similar for a range of pHi values in these experiments (and give that range).

Response: We have replaced the phrases in lines 128-130. Activities of wild-type and

mutant AE2 are recalculated in the same pHi range (at pHi 7.2) as suggested in line 365.

7. Previous studies of PIP2 regulation of NBCe1...Check that literature because they were not all patch-clamp studies.

Response: We have removed the reference to "patch-clamp" as suggested.

Reviewer #3 (Remarks to the Author):

The revised manuscript by Zhang et al is much improved and most of my suggestions and comments have been addressed. However, the manuscript remains challenging to read due to a large number of typos as well as grammar and syntax errors – many of which have been introduced during the revision. I therefore urgently recommend vigorous proofreading of the manuscript, maybe even by a third-party service provider.

Response: We thank Review #3 for the positive feedback. We have carefully proofread the manuscript and made considerable effort to improve syntax and grammar as requested.

References:

Xia, X., Liu, S., and Zhou, Z.H. (2022). Structure, dynamics and assembly of the ankyrin complex on human red blood cell membrane. Nat Struct Mol Biol 29, 698-705.

Zhang, Q., Jian, L., Yao, D., Rao, B., Xia, Y., Hu, K., Li, S., Shen, Y., Cao, M., Qin, A., et al. (2023). The structural basis of the pH-homeostasis mediated by the Cl(-)/HCO(3)(-) exchanger, AE2. Nat Commun 14, 1812.

REVIEWERS' COMMENTS

Reviewer #1 (Remarks to the Author):

The authors have conscientiously responded to the additional reviewer critiques. Only minor comments remain.

The data provided for review only concerning the thermal stability assay (the Superose elution profile pre-post heating at the different pH values, and the summary bar graph) should be presented as Supplemental Data.

Fig 4 legend or corresponding Results section should note that the bar graph represents 55% of AE2 input protein (pre-heating), but 80% of AE1 input protein, consistent with the different thermal stabilities. The additional text should also note that AE2 dimer in LMNG solution pH 7.4 is no more heat-stable than AE2 monomer, if not indeed less heat-stable (based on the Superose profile)

Point-by-point response to reviewers

Reviewer #1 (Remarks to the Author):

The authors have conscientiously responded to the additional reviewer critiques. Only minor comments remain.

The data provided for review only concerning the thermal stability assay (the Superose elution profile pre-post heating at the different pH values, and the summary bar graph) should be presented as Supplemental Data.
Fig 4 legend or corresponding Results section should note that the bar graph represents 55% of AE2 input protein (pre-heating), but 80% of AE1 input protein, consistent with the different thermal stabilities. The additional text should also note that AE2 dimer in LMNG solution pH 7.4 is no more heat-stable than AE2 monomer, if not indeed less heat-stable (based on the Superose profile)

Thank you for your review and suggestions. We have added the results of thermal stability assay in Supplementary Figure. 4f-h as requested and noted the residual fluorescences of AE1 and AE2 at pH 7.4 in the legend. The second peak of the superose profile does not represent AE2 monomer but GFP. We are not aware of the thermal stability of AE2 monomer at different pH.
